# Loss of tumor cell MHC class II drives MAPK inhibitor insensitivity of BRAF-mutant anaplastic thyroid cancers

**DOI:** 10.1172/JCI191781

**Published:** 2025-08-19

**Authors:** Vera Tiedje, Jillian Greenberg, Tianyue Qin, Soo-Yeon Im, Gnana P. Krishnamoorthy, Laura Boucai, Bin Xu, Jena D. French, Eric J. Sherman, Alan L. Ho, Elisa de Stanchina, Nicholas D. Socci, Jian Jin, Ronald A. Ghossein, Jeffrey A. Knauf, Richard P. Koche, James A. Fagin

**Affiliations:** 1 Human Oncology and Pathogenesis Program,; 2 Department of Medicine, and; 3 Department of Pathology and Laboratory Medicine, Memorial Sloan Kettering Cancer Center, New York, New York, USA.; 4 Department of Medicine, Division of Endocrinology, Metabolism, and Diabetes, and; 5 University of Colorado Cancer Center, University of Colorado Denver, Aurora, Colorado, USA.; 6 Antitumor Assessment Core Facility and; 7 Bioinformatics Core, Memorial Sloan Kettering Cancer Center, New York, New York, USA.; 8 Mount Sinai Center for Therapeutics Discovery, Departments of Pharmacological Sciences, Oncological Sciences, and Neuroscience, Tisch Cancer Institute, Icahn School of Medicine at Mount Sinai, New York, New York, USA.; 9 Lerner Research Institute, Cleveland Clinic, Cleveland, Ohio, USA.; 10 Center for Epigenetics Research, Memorial Sloan Kettering Cancer Center, New York, New York, USA.

**Keywords:** Endocrinology, Immunology, Antigen, Cancer, Thyroid disease

## Abstract

Cancer cells present neoantigens dominantly through MHC class I (MHCI) to drive tumor rejection through cytotoxic CD8^+^ T cells. There is growing recognition that a subset of tumors express MHC class II (MHCII), causing recognition of antigens by TCRs of CD4^+^ T cells that contribute to the antitumor response. We found that mouse *Braf^V600E^*-driven anaplastic thyroid cancers (ATCs) responded markedly to the RAF plus MEK inhibitors dabrafenib and trametinib (dab/tram) and that this was associated with upregulation of *MhcII* in cancer cells and increased CD4^+^ T cell infiltration. A subset of recurrent tumors lost *MhcII* expression due to silencing of *Ciita*, the master transcriptional regulator of *MhcII*, despite preserved IFN-γ signal transduction, which could be rescued by EZH2 inhibition. Orthotopically implanted *Ciita^–/–^* and *H2-Ab1^–/–^* ATC cells into immune-competent mice became unresponsive to the MAPK inhibitors. Moreover, depletion of CD4^+^, but not CD8^+^, T cells also abrogated the response to dab/tram. These findings implicate MHCII-driven CD4^+^ T cell activation as a key determinant of the response of *Braf*-mutant ATCs to MAPK inhibition.

## Abstract 2

We provide evidence that the response of *BRAF*^V600E^ mutant anaplastic thyroid cancer to MAPK is primarily immune-mediated and that one mechanism of resistance is loss of MHC Class II expression by epigenetic silencing of *Ciita*.

## Introduction

Antigen presentation, processing, and consequent T cell priming are essential for an effective immune-mediated antitumor response. Alterations in the antigen presentation pathway in tumor cells are well described immune escape mechanisms in patients resistant to checkpoint inhibitor therapy (1–3). Intracellular peptides are presented by MHC class I (MHCI) to CD8^+^ T cells, whereas extracellular peptides are presented by MHC class II (MHCII) molecules to CD4^+^ T cells. MHCI is ubiquitously expressed by nucleated cells, whereas MHCII is primarily expressed by professional antigen-presenting cells such as dendritic cells (2). Some tumor cell lineages, such as breast (4), melanoma, (5) and lung (6), express MHCII when exposed to IFN-γ. IFN-γ leads to JAK1/2 and STAT1 phosphorylation; STAT1 in turn translocates to the nucleus and cooperates with IRF1 to activate the pIV promoter of the gene encoding MHCII transactivator (*CIITA*), which functions as a scaffold for the RFX family members *RFX5*, *RFXAP*, and *RFXANK* to drive transcription of MHCII-related genes (reviewed in ref. 7).

Although the presentation of tumor neoantigens through MHCI to CD8^+^ T cells is fundamental to the immune response to cancer (8–10), CD4^+^ T cells can also eradicate tumors in an antigen-dependent manner in tumor cells that express MHCII (11, 12). Studies in mice implanted with B16 melanoma cells expressing either the Trp1 or pMel model antigens and infused with CD4^+^ and CD8^+^ transgenic T cells that recognize these individually or in combination have helped delineate the contribution of MhcI and MhcII antigen presentation to antitumor immune responses (13).

A pooled human kinome shRNA screen of mesothelioma cells identified *RET*, *MAPKK* (*MEK*), and *ERK* as negative regulators of cell-surface abundance of HLA-A02:01, which plays a central role in antigen presentation by MHCI (14), a finding confirmed in other cancer cell lineages. This implicates oncogenic activation of the MAPK pathway in suppressing the antigen presentation machinery driving CD8^+^ T cell activation. In papillary thyroid cancer (PTC), tumor cell–specific MHCII expression is suppressed in *BRAF^V600E^*-driven tumors through a TGF-β–dependent autocrine loop (15) and rescued by MAPK pathway inhibitors. *BRAF^V600E^* is also the most common MAPK pathway driver in anaplastic thyroid cancer (ATC), commonly associated with mutations of the *TERT* promoter, *TP53*, and genes that regulate chromatin remodeling (16, 17). ATC is a devastating disease with a median overall survival that until recently rarely exceeded 6 months. Combined chemoradiation improves survival modestly for stage IVA/B ATC as compared with palliative therapy but provides no benefit to patients with distant metastatic disease (18). A phase II study of *BRAF^V600E^*-mutant ATC treated with the RAF kinase inhibitor dabrafenib and the MEK inhibitor trametinib (dab/tram) showed a remarkable 56% overall response rate (ORR) (19, 20). By contrast, the ORR of *BRAF^V600E^*-driven differentiated TC (DTC) to dab/tram was only 30% (21). This was unexpected, as ATCs have a more disrupted genome and aggressive behavior. The tumor microenvironment (TME) of ATCs, as compared with DTCs, is characterized by much heavier infiltration with macrophages and myeloid-derived suppressor cells (MDSCs) and enrichment for NK, CD4^+^, and CD8^+^ T cells (22–24). Overall survival was also improved in ATC patients; however, most patients eventually progressed (20, 25). We previously showed in a murine model of ATC driven by thyrocyte-specific doxycycline-inducible (dox-inducible) *BRAF^V600E^* in the context of *Tp53* loss that tumors regress profoundly upon BRAF inhibition but often recur. A common mechanism of recurrence is development of *Met* amplification associated with overexpression of its ligand, *Hgf*, resulting in reactivation of MAPK signaling (26). A similar resistance mechanism has been documented in patients with ATC with secondary resistance to BRAF/MEK inhibition (27).

Although reactivation of the MAPK pathway is a recognized mechanism of resistance to MAPK pathway inhibitors in several tumor types, the role of tumor cell–autonomous expression of components of the antigen presentation machinery in the response to these targeted therapies has not to our knowledge been investigated in vivo. This is pertinent to ATCs, because their rich inflammatory tumor microenvironment predicts that the response to MAPK inhibitors is in part immune mediated. We found epigenetic silencing of *Ciita* in a subset of recurrent mouse ATC following BRAF inhibition, which led us to investigate the role of MhcII and CD4^+^ T cells in the response to dabrafenib and trametinib (dab/tram). We show that *Ciita^–/–^* or *H2-Ab1^–/–^* ATCs were resistant to dab/tram and that CD4^+^ T cells were required for the response to these drugs.

## Results

### Human and murine ATCs are heavily infiltrated by T cells and macrophages.

**Figure 1:**
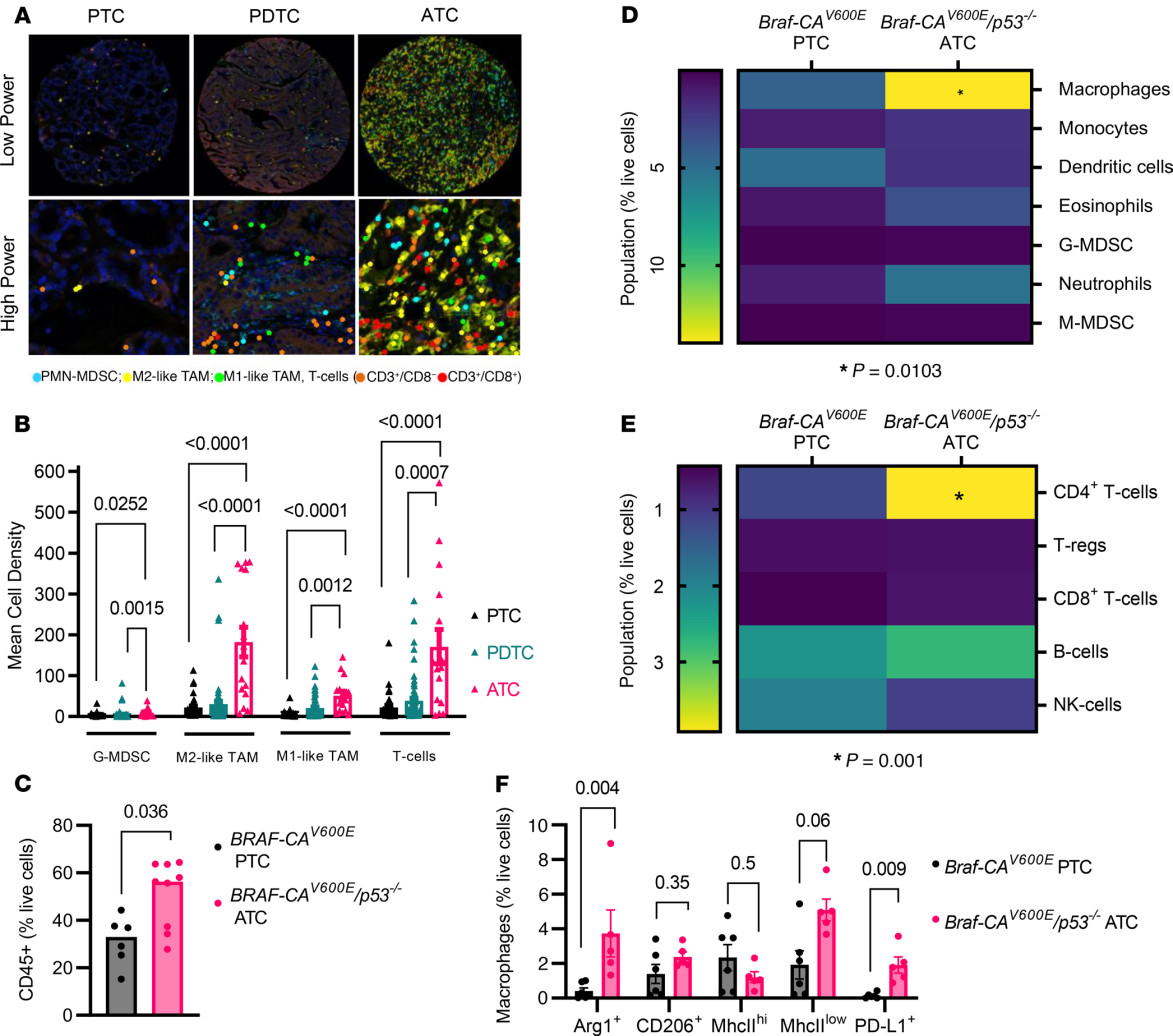
Composition of the tumor immune microenvironment in human and murine thyroid cancers. (**A**) Representative images of multiplex immunofluorescence staining for CD3, CD8, CD68, CD163, and CD15 of TMAs of 41 PTCs, 72 PDTCs, and 16 ATCs. Original magnification: Low power, 200µm; high power, 50 µm. (**B**) Quantification of TMA for G-MDSC (CD15^+^), M1-like TAM (CD68^+^/CD163^–^), M2-like TAM (CD68^+^/CD163^+^), and T cells (CD3^+^/CD8^–^ and CD3^+^/CD8^+^). (**C**–**E**) Characterization of immune TME of murine *Braf-CA^V600E^* PTCs (*n* = 6) and *Braf-CA^V600E^/p53^–/–^* ATCs (*n* = 9) by multispectral flow cytometry: (**C**) CD45^+^ cells. (**D**) Myeloid subpopulations including macrophages (CD11b^+^Ly6G^–^Ly6C^–^F480^+^), monocytes (CD11b^+^Ly6G^–^Ly6C^+^), dendritic cells (CD11c^+^F480^–^MhcII^+^), eosinophils (CD11b^+^Ly6C^–^Ly6G^+^Siglec F^+^), G-MDSC (CD11b^+^Ly6G^+^Ly6C^–^Arg1^+^), neutrophils (CD11b^+^Ly6G^+^Ly6C^–^Arg1^–^), and M-MDSC (CD11b^+^Ly6G^–^Ly6C^+^Arg1^+^). (**E**) Lymphoid cells including CD4^+^ T cells (CD3^+^CD4^+^FoxP3^–^), Tregs (CD3^+^ CD4^+^ FoxP3^+^), CD8^+^ T cells (CD3^+^ CD8^+^), B cells (B220^+^) and NK cells (B220- NK1.1^+^). (**F**) Macrophage subtypes. **B**, **C**, and **F**: Multiple Mann-Whitney tests; **D** and **E**: 2-sided ANOVA with Šidák’s multiple-comparison test, with single pooled variance. Data are presented as mean ± SEM. TMA, tissue microarray; PDTC, poorly differentiated thyroid cancer; G-MDSC: granulocytic MDSCs; TAM, tumor-associated macrophages; M-MDSC, monocytic myeloid-derived suppressor cells.

We characterized the TME of human PTCs, poorly differentiated thyroid cancers (PDTCs), and ATCs with multiplex immunofluorescence histochemistry on tissue microarrays (Figure 1A). Among these tumor types, ATCs were the most heavily immune-infiltrated, predominantly with macrophages and T cells (Figure 1B). Consistent with this, multispectral flow cytometry of a murine genetically engineered mouse model (GEMM) of ATC (*Tpo-Cre/eYFP/BRaf-CA/Trp53f^l/fl^*; termed *Braf-CA^V600E^/p53*) showed a greater proportion of CD45^+^ cells in relation to live cells than a GEMM of PTC (*Tpo-Cre/Braf-CA^V600E^*; termed *Braf-CA^V600E^*) (Figure 1C). The greater myeloid population of ATCs as compared with PTCs primarily consisted of macrophages (CD11b^+^Ly6C^–^Ly6G^–^ F480^+^) (Figure 1D), and their lymphoid infiltrate was particularly enriched for FoxP3^–^ CD4^+^ T cells (Figure 1E). Further subtyping of the macrophage population showed a more immunosuppressive phenotype in ATCs than PTCs, characterized by arginase-1– (Arg1-) and CD206-positive cells with low MhcII expression, as well as a higher proportion of PD-L1–expressing macrophages (Figure 1F).

Human and mouse *BRAF^V600E^*-driven ATCs have a higher MAPK transcriptional output than PTCs (26). Moreover, the mutational burden of ATCs as determined by whole exome sequencing (WES) was comparable in the 2 species (Supplemental Table 1; supplemental material available online with this article; https://doi.org/10.1172/JCI191781DS1) (26, 28, 29). Hence, the data indicated that GEMMs of ATC closely phenocopy their human counterparts and represent a valid model of the disease.

### MAPK pathway inhibition in mouse Braf^V600E^/p53^–/–^ ATC tumor cells activates IFN-γ transcriptional output and expression of genes in the antigen presentation pathway.

**Figure 2:**
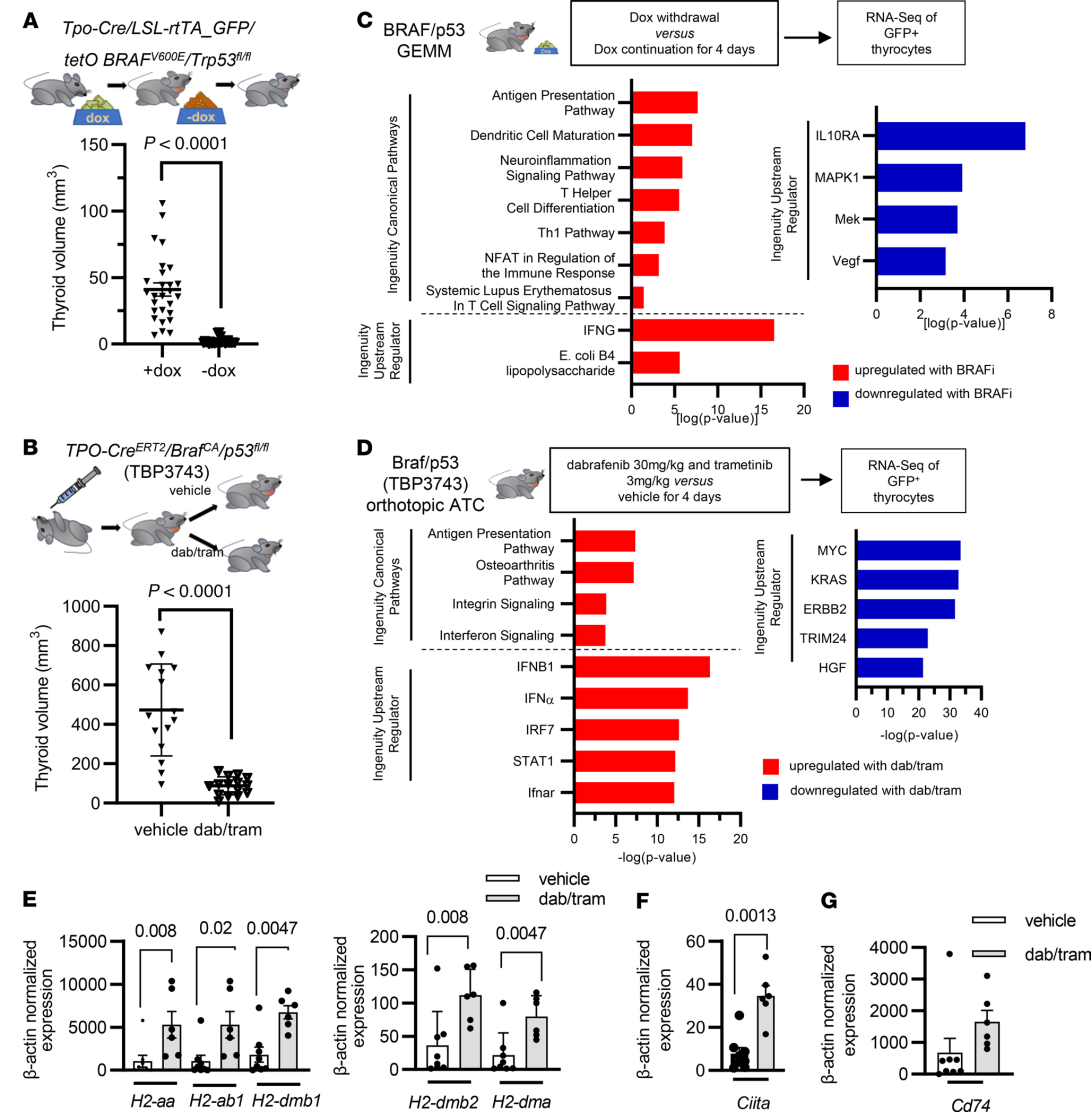
Induction of antigen presentation pathways in *BRAF^V600E^* ATC in response to MAPK inhibition. Thyroid volume measurements (**A**) by MRI of dox-inducible BRAF/p53 GEMM on (*n* = 27) and off (*n* = 27) dox for 3–4 weeks and (**B**) by ultrasound in mice orthotopically injected with TBP3743 cells and treated 1 week after engraftment with 30 mg/kg dabrafenib and 3 mg/kg trametinib (*n* = 15) or vehicle (*n* = 15) for 2 weeks. Ingenuity pathway analysis of sorted thyrocytes of BRAF/p53 ATCs on and off dox (**C**) and orthotopic Braf/p53 ATCs treated with vehicle or dab/tram for 4 days (**D**). (**E**–**G**) RT-PCR of the indicated MhcII complex mRNAs, Ciita, and Cd74 in sorted thyrocytes from Braf/p53 orthotopic ATCs treated with vehicle or dab/tram in vivo for 4 days. **A**, **B**, and **E**–**G**: Multiple Mann-Whitney tests. Data are presented as mean ± SEM. GEMM, genetically engineered mouse model.

Patients with *BRAF^V600E^*-driven ATCs show remarkable structural responses to combined RAF and MEK inhibitors, to the extent that this enables neoadjuvant surgical resection in a subset of patients initially presenting with unresectable disease (25). We used 2 models to investigate the transcriptional responses of mouse *Braf^V600E^*-mutant ATCs to inhibition of the driver: (i) mice with thyroid-specific doxycycline-inducible (dox-inducible) expression of BRAF^V600E^ in the context of *Tp53* loss, which included a GFP fluorescent reporter for cell tracking (*Tpo-Cre/LSL-rtTA_GFP/tetO-mycBRAF^V600E^/p53^fl/fl^* (BRAF/p53) (26); and (ii) immune-competent mice with orthotopic implantation of the *Braf^V600E^/p53^–/–^* cell line TBP3743 (Braf/p53) into the thyroid (30). As previously reported, the BRAF/p53 ATCs showed profound responses to BRAF inhibition by dox withdrawal (Figure 2A). Combination therapy with dab/tram also resulted in marked tumor shrinkage of orthotopic ATCs (Figure 2B) and substantially improved survival (Supplemental Figure 1A).

To understand the transcriptional changes associated with these profound responses to therapy, we performed bulk RNA-Seq of FACS-sorted thyrocytes from both models. Ingenuity pathway analysis identified the top differentially up- and downregulated pathways in the context of either BRAF inhibition by dox withdrawal in BRAF/p53 ATCs or dab/tram treatment in the orthotopic Braf/p53 TBP3743 model (Figure 2, C and D). Canonical pathways and upstream regulators enriched by MAPK inhibition in both contexts were related to antigen presentation and interferon pathway activation and, in the BRAF/p53 model, to CD4^+^ T helper cell maturation.

To further delineate the impact of the MAPK pathway on tumor cell antigen presentation, we investigated expression of genes of the MhcI and -II pathways in FACS-sorted thyrocytes from WT thyroids and TBP3743 ATCs treated with vehicle or dab/tram for 4 days. MhcI in mice is encoded by the *B2m*, *H2-k1*, and *H2-d1* genes, whereas MhcII is encoded by *H2-aa*, *H2-ab1*, *H2-dma*, *H2-dmb1*, and *H2-dmb2*. MhcII-related genes were expressed at low levels in orthotopic TBP3743 and BRAF/p53 GEMM ATCs and markedly induced by dox withdrawal and dab/tram treatment, respectively (Figure 2E and Supplemental Figure 1B). Interestingly, MhcII genes were also expressed in WT thyrocytes, with levels intermediate between those of BRAF/p53 ATCs prior to and after dox withdrawal (Supplemental Figure 1B). Rfx5, Rfxap, and Rfxank are DNA-binding proteins that cooperate with Ciita to activate transcription of MhcII-related genes. However, only the expression of *Ciita,* but not that of *Rfx* genes, was markedly induced by MAPK pathway inhibition (Figure 2F and Supplemental Figure 1, C and D). *Cd74*, which is transcriptionally dependent of *Ciita* and assembles with the MhcII alpha and beta chains in the endoplasmic reticulum (ER) to prevent endogenous peptide binding, was suppressed in vehicle-treated ATCs and rescued by MAPK inhibition (Figure 2G and Supplemental Figure 1E). By contrast to MhcII genes, expression of the MhcI genes *H2-k*, *H2-d*, and *B2m* were only modestly suppressed in Braf-ATC versus WT thyroid cells, with expression induced by MAPK inhibition (Supplemental Figure 1, F–H).

### Recurrent human and murine BRAF-driven ATCs show attenuated MhcII expression in response to MAPK pathway inhibition.

**Figure 3:**
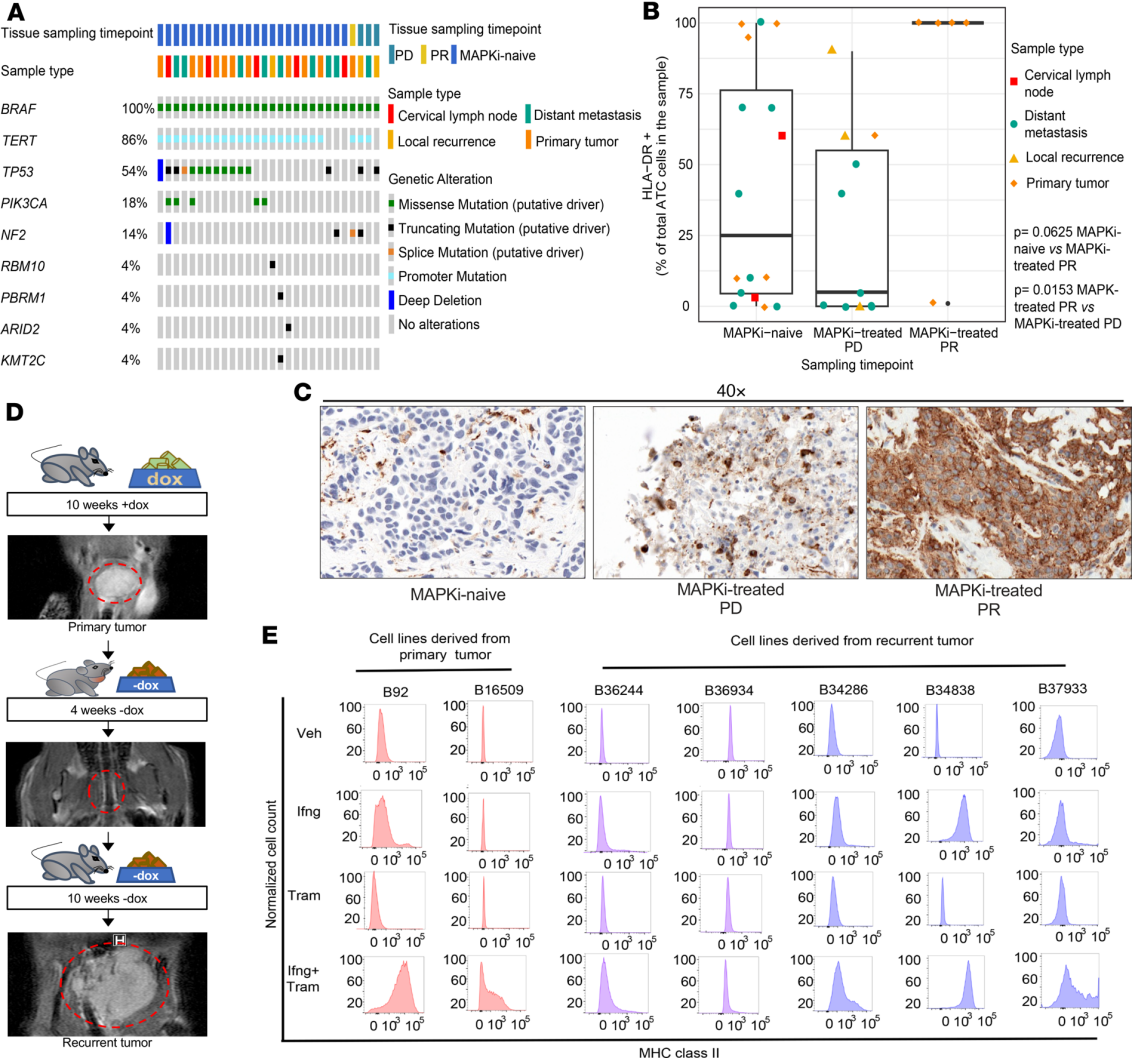
MhcII expression is suppressed in recurrent ATCs. (**A**) Oncoprint of ATC specimens from 28 patients from the Memorial Sloan Kettering clinical cohort sequenced by MSK-IMPACT and subjected to HLA-DR IHC. (**B**) Percentage of ATC cells expressing HLA-DR in specimens from patients prior to MAPK inhibitor therapy (MAPKi naive, *n* = 17), at the time of structural response (MAPKi PR, *n* = 5), or during disease progression (MAPKi PD, *n* = 10) under therapy with dab/tram (*n* = 22), vemurafenib (*n* = 1), dabrafenib (*n* = 1), or PLX8394 (*n* = 1). (**C**) Representative IHC images of HLA-DR IHC. (**D**) Representative MRIs of a primary ATCs (+dox), the response 4 weeks after dox withdrawal (-dox), and a recurrence at 10 weeks (–dox). (**E**) MhcII flow cytometry of cell lines generated from primary ATCs (B92 and B16509) and recurrent tumors (B36244, B36934, B34286, B34838, and B37933) treated for 96 hours with vehicle (Veh), IFN-γ (20 ng/mL), trametinib (10 nM), or IFN-γ + trametinib in vitro. Plasma membrane MhcII levels were not induced by IFN-γ + trametinib in the recurrent ATC cell lines B36244 and B36934. **B**: Multiple Mann Whitney Tests. Mean with SEM. Bounds of the boxes represent interquartile ranges (IQR), interior lines represent the median, whiskers extend to minimum and maximum values, and outlying dots represent values 1.5 times the IQR. MAPKi, MAPK inhibitor; PR, partial response; PD, progressive disease.

To determine whether MHCII expression was associated with response to MAPK inhibitor therapy, we performed IHC for HLA-DR in *BRAF^V600E^*-driven ATC samples from untreated patients, from those showing partial response to or stable disease with this therapy, and from patients with progressive disease (Figure 3A and Table 1). HLA-DR expression by ATC cells was dampened in specimens from MAPK inhibitor–naive patients (Figure 3B). By contrast HLA-DR was expressed by all ATC cells in 4 of 5 surgical samples from patients with a structural response to BRAF and/or MEK inhibitor treatment, whereas markedly fewer ATC cells expressed HLA-DR in specimens from progressive lesions (Figure 3C).

Although murine BRAF/p53 ATCs shrank dramatically upon dox withdrawal, most of them ultimately recurred (Figure 3D). We previously reported that the recurrent tumors were characterized by *BRAF^V600E^*-independent mechanisms of MAPK pathway reactivation, such as *Met* gene amplification and *Ras* point mutations, which were detected in a subset of the recurrences (26). We generated cell lines from primary (B92, B16509) and recurrent tumors (B36244, B37933, B34286, B36934, B34838). The B36934 and B34838 lines were derived from *Met*-amplified recurrences.

**Figure 4:**
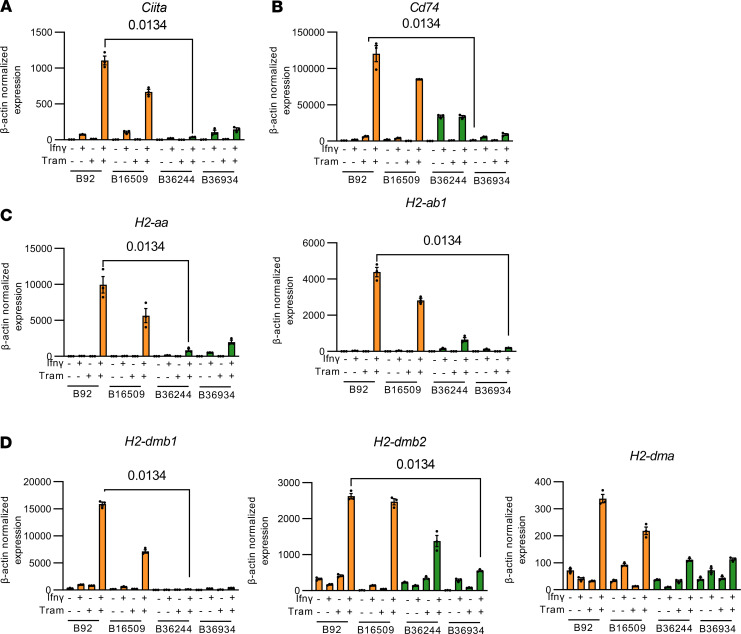
Ciita expression is lost in recurrent ATC cells. (**A**–**D**) RT-PCR of 2 primary and 2 “MhcII-low” recurrent cell lines for the indicated MhcII complex genes (**C** and **D**), *Ciita* (**A**), and *Cd74* (**B**) in response to IFN-γ, trametinib, or IFN-γ + trametinib for 96 hours. Kruskal-Wallis test. Data are presented as mean ± SEM.

Since transcriptional induction of antigen presentation pathways, primarily of MhcII, was a key feature of response of mouse ATCs to MAPK inhibition, we next examined whether this response was retained in cell lines derived from recurrent tumors. We determined MhcI and II expression by flow cytometry in 2 primary and 5 recurrent cell lines in response to DMSO, IFN-γ (20 ng/mL), trametinib (10 nM), or the combination of IFN-γ and trametinib for 96 hours. Whereas IFN-γ was sufficient to induce MhcI expression in both primary and recurrent cell lines (Supplemental Figure 2A), the combination of IFN-γ and trametinib was required to maximally induce MhcII in cell lines from the primary ATCs (Figure 3E and Figure 4, C and D). MhcII expression was not rescued by IFN-γ and trametinib in 2 of the 5 recurrent cell lines, B36244 and B36934 (Figure 3E and Figure 4, A and B). This was not due to impaired inhibition of MAPK by trametinib or activation of IFN-γ downstream signaling (Supplemental Figure 2B). Notably, expression of *Ciita* and *Cd74* was markedly attenuated in the 2 recurrent as compared with the 2 primary cell lines (Figure 4, C and D).

### Loss of MhcII expression in recurrent ATCs is due to epigenetic silencing of Ciita.

**Figure 5:**
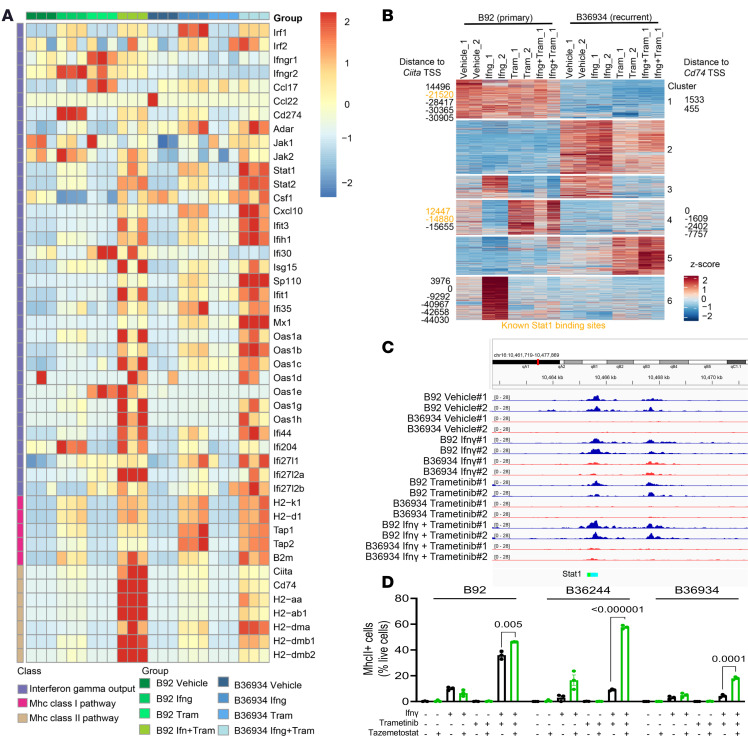
Loss of tumor cell MhcII expression is associated with epigenetic silencing of the Ciita locus. (**A**) RNA-Seq of B92 and B36934 cells, highlighting MhcI-, MhcII-, and IFN-γ–related gene expression pathways in response to a 96-hour treatment with vehicle, IFN-γ, trametinib, or IFN-γ + trametinib. (**B**) Unsupervised *k*-means clustering of ATAC-Seq peak gains (red) and losses (blue) in B92 and B36934 cells in the indicated treatment conditions. (**C**) Integrative Genomics Viewer (IGV) plot showing ATAC-Seq peak losses in the recurrent cell line B36934 in comparison to the primary cell line B92 at Stat1 transcription factor binding sites related to the *Ciita* locus. (**D**) Percentage of live cells expressing MhcII as determined by FACS in B92 and B36934 cells treated with IFN-γ, trametinib, or IFN-γ + trametinib, with or without addition of the EZH2 inhibitor tazemetostat.

To probe into the underlying mechanisms of MhcII loss and attenuated *Ciita* expression in recurrent versus primary cell lines, we performed bulk RNA-Seq (GSE302631) of a primary (B92) and recurrent (B36934) cell line after a 96-hour treatment with DMSO, IFN-γ (20 ng/mL), trametinib (10 nM), or the combination of IFN-γ and trametinib. It is important to note that these are not isogenic cell lines and that principal component analysis (PCA) analysis of their transcriptomes showed that they were distinct from each other at baseline (Supplemental Figure 3, A and B). Because of that, we focused our analysis on a previously defined IFN-γ transcriptional output (31) and on the genes required for expression of the MhcI and -II complexes. Consistent with our flow cytometry data (Figure 3E), the response to IFN-γ was markedly attenuated in both cell lines and rescued by cotreatment with trametinib (Figure 5A). The MhcII pathway was robustly induced by combination therapy only in the primary cell line (Figure 5A).

Since there was no apparent global impairment of IFN-γ signaling or transcriptional output between primary and recurrent cell lines (Supplemental Figure 2B), we tested whether silencing of *Ciita* was associated with decreased chromatin accessibility by ATAC-Seq (GEO GSE302631). The ATAC-Seq dynamic peaks in the B92 and B36934 lines were resolved into 6 clusters (Figure 5B). We mapped the peaks that were within 50 kb of the transcription start sites (TSS) of *Ciita* and *Cd74* and found that these were confined to clusters 1, 4, and 6 (Figure 5B). Chromatin accessibility in these clusters was decreased in the recurrent cell line in all treatment conditions. We integrated our ATAC-Seq data with the ChIP-Atlas data (32, 33) and found that multiple ATAC peaks around *Ciita* were genuine binding sites for STAT1 and were exclusively accessible in the cell line derived from the primary tumor (Figure 5, C). Reflecting the findings from the gene expression analysis, global analysis of *Stat1* and *Irf1* binding sites based on previously documented data in IFN-γ–stimulated macrophages did not show differences between the 6 clusters defined by our ATAC-Seq data (32, 33) (Supplemental Figure 4). We used HOMER motif analysis to identify transcription factor motif enrichments in the 6 ATAC clusters. Motifs related to transcriptional regulators of MhcII genes such as Stat and Rfx family members were enriched in clusters 1, 4, and 6 (Supplemental Figure 3, C–E).

*Ciita* expression has been reported to be dependent on BRG1 (SMARCA4), one of the 2 mutually exclusive ATPases that serve as catalytic subunits of SWI/SNF chromatin remodeling complexes. Upon IFN-γ stimulation, BRG1 displaces EZH2 and SUZ12, key components of polycomb repressor complex 2 (PRC2), from inter-enhancer regions across the *Ciita* locus (34). As loss-of-function mutations of SWI/SNF genes are common in advanced thyroid cancers (17, 35), we performed WES on B34286 cells, which retained IFN-γ– and trametinib-induced MhcII expression, and on the 2 recurrent cells that did not (B36934 and B36244), using tail DNA from the corresponding mouse line as control, and found no nonsynonymous mutations in any of the Swi/Snf genes (Supplemental Table 2; GEO GSE301723). To test whether PRC2 activity accounted for the repression of MhcII in the 2 recurrent cell lines we treated them with tazemetostat, a catalytic EZH2 inhibitor. Pretreatment of B92, B36934, and B36244 cells with tazemetostat for 96 hours, followed by IFN-γ trametinib for a further 48 hours, either completely or partially rescued MhcII in the recurrent lines (Figure 5D).

### Response to MAPK inhibition in BRAF^V600E^/p53^–/–^ ATCs requires tumor cell MhcII expression and is CD4^+^ T cell dependent.

**Figure 6:**
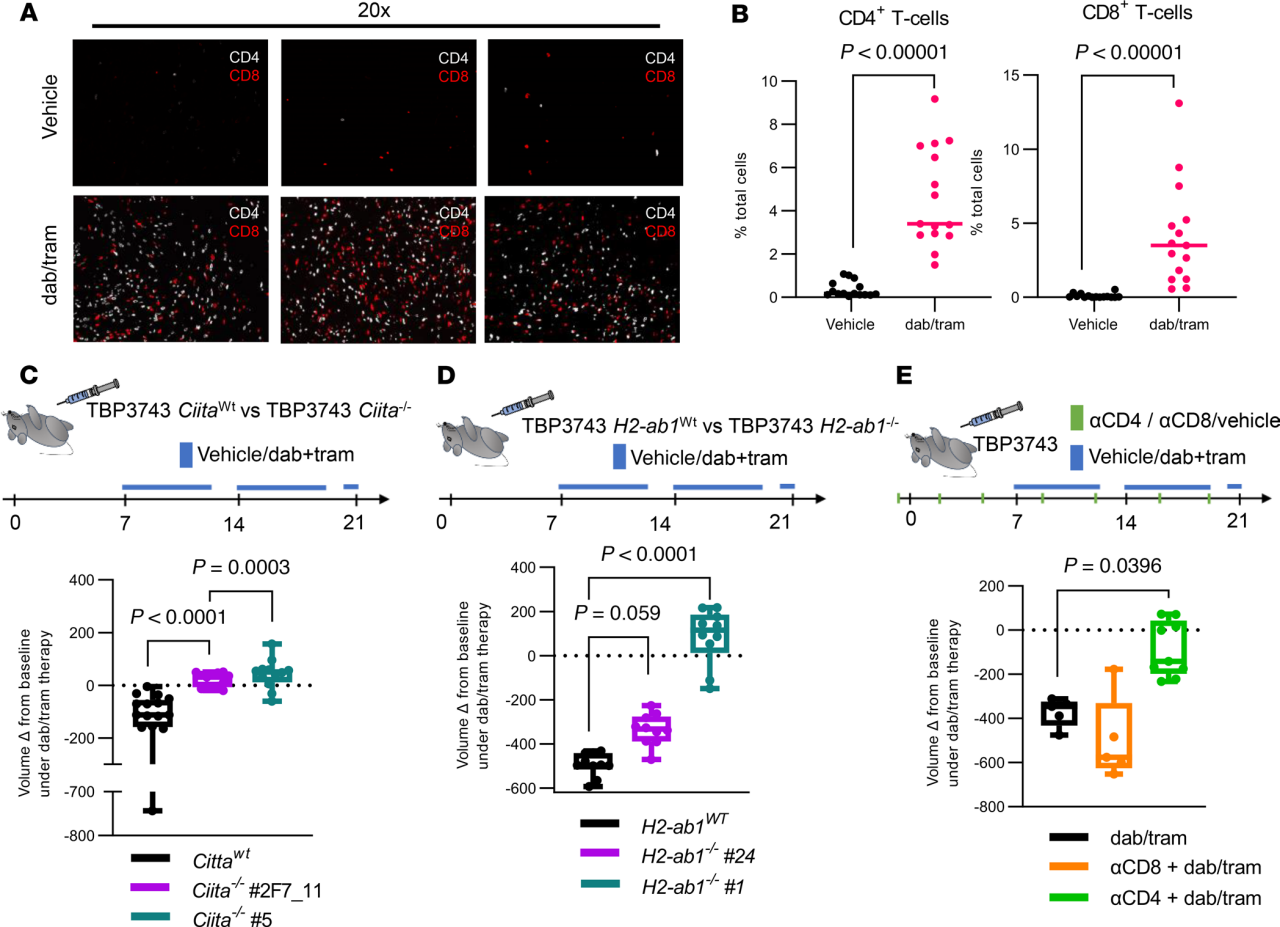
Response of *BRAF^V600E^*-driven ATCs to dab/tram is lost in *Ciita^–/–^* and *H2-ab1^–/–^* ATCs and is CD4^+^ T cell dependent. (**A**) Representative CD4 and CD8 immunofluorescence of Braf/p53 ATCs treated with vehicle or dab/tram for 10 days. (**B**) Quantification of CD4^+^ and CD8^+^ T cells in ATC slides as determined by immunofluorescence. Each dot represents an individual tissue specimen; *n* = 15 for each treatment condition. (**C**) Tumor volume changes of *Ciita^wt^* (*n* = 16) and 2 clones (*n* = 13 for #2F7_11 and *n* = 12 for #5) of *Ciita^–/–^* TBP3743 cells in response to dab/tram for 2 weeks. (**D**) Response of *H2-ab1^wt^* (*n* = 10) and 2 clones of *H2-ab1^–/–^* (*n* = 10 for #24 and *n* = 11 for #1) to dab/tram treatment for 2 weeks. (**E**) Tumor volume change of orthotopic ATCs in response to treatment with dab/tram (*n* = 9) or in combination with α-CD4 (*n* = 6) or α-CD8 (*n* = 5) depletion antibody. **B**: Multiple Mann-Whitney tests. **C**–**E**: Kruskal-Wallis test. Data are presented as mean ± SEM. Whiskers indicate minimum to maximum and bounds of the boxes represent interquartile ranges.

Orthotopically implanted TBP3743 cells become heavily infiltrated by both CD4^+^ and CD8^+^ T cells in response to dab/tram (Figure 6, A and B), but the relative contribution of these 2 T cell populations to the response to MAPK inhibition is unknown. We also observed increased macrophage infiltration with a markedly smaller fraction of Arg1^+^ and CD206^+^ macrophages, suggesting a less immunosuppressive environment (Supplemental Figure 5, A and B). Because recurrent mouse ATCs can lose MhcII, we asked whether silencing of *Ciita* and MhcII expression was sufficient to induce resistance to dab/tram. For this, we generated homozygous CRISPR-KO clones of *Ciita* and *H2-Ab1*, a major component of the MhcII complex, in TBP3743 cells (Supplemental Figure 5, C–E). We confirmed that MhcII expression as measured by FACS was absent in both *Ciita^–/–^* and *H2-Ab1^–/–^* clones (Supplemental Figure 5, F and G). We generated orthotopic models with *Ciita^–/–^* and *H2-Ab1^–/–^* ATCs, treated the mice with dab/tram (schema in Figure 6, C and D), and performed serial ultrasounds to assess therapy response as determined by tumor volume change. Whereas the IC_50_ to trametinib of 2 independently derived clones of *Ciita^–/–^* and *H2-Ab1^–/–^* was comparable to that of the parental cell line in vitro (Supplemental Figure 6, A and C), *H2-Ab1^–/–^* and *Ciita^–/–^* TBP3743 cells showed attenuated responses to dab/tram in vivo (Figure 6, C and D, and Supplemental Figure 6, B and D). Moreover, depletion of CD4^+^, but not CD8^+^, T cells impaired the response to dab/tram (Figure 6E and Supplemental Figure 6, E–G).

## Discussion

**Figure 7:**
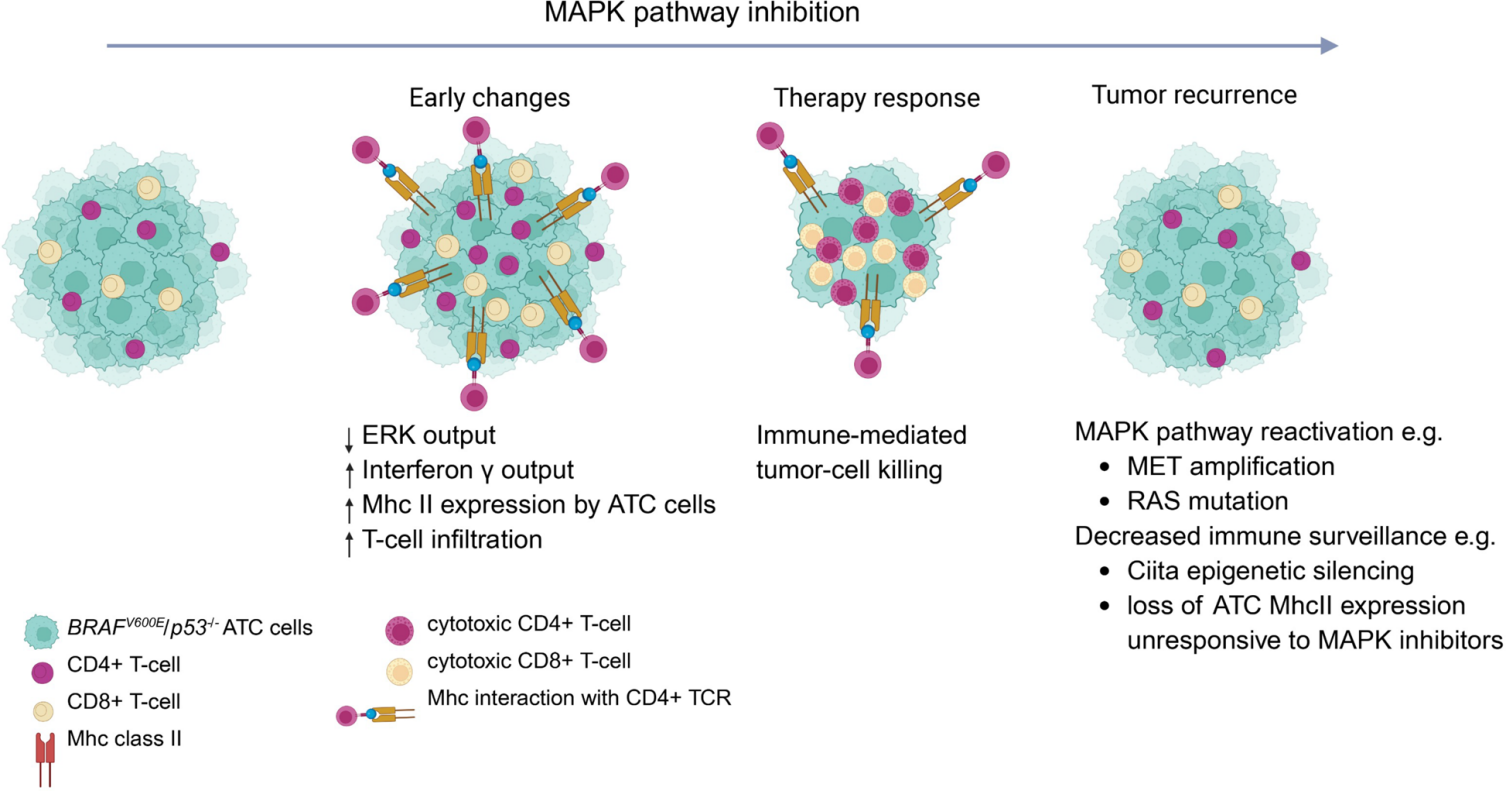
Mechanisms of response and recurrence to MAPK pathway inhibition in BRAF^V600E^/ p53^–/–^ ATC. Upon MAPK inhibition, there is an induction of IFN-γ transcriptional output in ATC cells, leading to MhcII expression and T cell–mediated tumor cell death. Recurrences can occur through MAPK pathway reactivation and/or decreased immune surveillance due to Ciita silencing and loss of MhcII expression.

In this study, we showed that *Braf*-mutant ATC tumor cells markedly activated antigen presentation programs in response to therapies targeting the oncoprotein and that induction of MHCII expression was a particularly determinant factor in response to therapy. The most persuasive evidence supporting this was the discovery that combined treatment of ATC cell lines derived from recurrent tumors with trametinib and IFN-γ failed to induce MHCII, and that this was due to epigenetic silencing of *Ciita*. Moreover, knockdown of either *Ciita* or *H2-ab1*, or depletion of CD4^+^ T cells, was sufficient to abrogate response to MAPK inhibitors in vivo. This implicates modulation of the interaction of tumor cells with the immune microenvironment as a key mechanism of response to dabrafenib and trametinib in *Braf*-mutant ATC (Figure 7). A prior study reported that treatment of *BRAF*-mutant PTC cell lines with MAPK inhibitors induced MHC2 expression through a TGF-β autocrine loop (15), but the implications of this finding to resistance mechanisms had not to our knowledge been previously explored.

There are parallels to these findings in *BRAF*-mutant melanomas. Treatment of human melanoma cell lines with the RAF inhibitor vemurafenib restores expression of melanoma antigens, which are recognized by antigen-specific T cells (36). Moreover, treatment with MAPK inhibitors resulted in marked tumor infiltration by both CD4^+^ and CD8^+^ T cells after 7 days (37), consistent with our findings in the murine ATC model. Interestingly, a clinical trial of the combination of the MEK inhibitor cobimetinib with vemurafenib showed an increase in proliferating CD4^+^ T helper cells, with no increase in Tregs (38).

Resistance to therapies targeting oncogenic BRAF in murine and human thyroid cancers arises in part due to reactivation of MAPK signaling through mutations of other genes in the pathway, such as *KRAS*, *HRAS*, *RAC1*, and *NF1*, or through copy number abnormalities leading to *MET* gene amplification (26, 27, 39). Interestingly, the mouse ATC cell line B36934, derived from a recurrent tumor, harbors a *Met* gene amplification (26) as well as silencing of *Ciita* and refractoriness to induction of Mhc2 by combined treatment with trametinib and IFN-γ. Hence, these 2 distinct mechanisms of resistance can coexist.

There was a relative attenuation of the IFN-γ transcriptional output of *BRAF*-mutant ATC cells, which was rescued by MAPK inhibition in both primary and recurrent cell lines. Although there was some heterogeneity in the expression of individual IFN-γ–regulated genes, the most manifest difference between the index primary and recurrent cell lines was the absence of induction of the MhcII mRNA cluster in the latter, rather than global impairment of the entire IFN-γ cistrome. Accessibility of transcription factors to the *Ciita* locus is dependent on the integrity of Swi/Snf chromatin remodeling complexes (34). Indeed, IFN-γ–induced *Ciita* transcription is BRG1 (SMARCA4) dependent (34), conferred through eviction of the polycomb repressor complex 2 (PRC2) including its EZH2 and SUZ12 subunits. Dysregulated EZH2 recruitment to *Ciita* was identified as the underlying mechanism for the absence of MhcII expression in breast cancer cell lines (40). A PRC2 gene expression signature was also shown to be inversely associated with MhcII-related gene expression and T cell gene signatures in malignant melanoma (41). Moreover, EZH2 inhibition in MhcII-negative melanoma cell lines resulted in open chromatin at the IFN-γ–inducible promoter pIV of the *Ciita* gene and increased MhcII expression (41). Consistent with this, we also found that EZH2 inhibition led to partial MhcII rescue in ATC cell lines derived from recurrent tumors. Tumor cell MHCII expression has been proposed as a predictor of response to checkpoint inhibitor therapy (5), but to our knowledge, prior to our study, its loss had not been identified as an acquired resistance mechanism to MAPK inhibitors.

It is important to note that the impact of MAPK activation on MHC II expression may be lineage specific. For instance, ERK2 knockout in a human glioblastoma cell line was shown to suppress MHCII as compared with isogenic parental *BRAF^V600E^* mutant cells (42). Moreover, in glioblastoma patients, higher tumor cell ERK1/2 phosphorylation is associated with better outcomes in response to PD-1 inhibitors and with a higher CD8^+^ T cell infiltrate (42, 43).

Immunotherapy with combined neutralizing antibodies to PD-1 (nivolumab) and CTLA4 (ipilumimab) is the first line therapy for patients with metastatic *BRAF*-mutant melanoma (44), with a remarkable overall survival at 10 years of 52% for the combination, and 37% for nivolumab monotherapy (45). In the only single-agent anti–PD-1 inhibitor (spartalizumab) trial performed so far in ATC, the ORR was 19% and the median OS 5.9 months (46). A combination trial of ipilumimab and nivolumab for patients with thyroid cancer showed a 30% (3 of 10) ORR in the ATC cohort, with some of these responses being quite durable (47). Other preliminary small studies using anti–PD-1 and anti–CTLA-4 combinations reported at conferences show activity in a similar proportion of ATC patients (48, 49). Importantly, when the PDL-1 inhibitor atezolizumab was combined upfront with RAF + MEK inhibitors (vemurafenib and cobimetinib), the median OS was 43 months (50), as compared with the historical 14.5-month median OS in response to targeted therapy with MAPK inhibitors alone (20). Taken together, these data indicate that, by contrast to melanomas, primary treatment with immune checkpoint inhibitor (ICI) commonly fails to overcome the immune-suppressive microenvironment of BRAF-mutant ATC. Interestingly, sequential therapy with MAPK inhibitors followed by anti–PD-1 and anti–CTLA-4 was inferior to upfront immunotherapy in terms of disease progression in patients with melanoma (51). However, the immune TME in ATC is distinct from that of melanomas. As treatment with MAPK inhibitors promoted a more immunogenic TME in Braf-ATC, these data suggest that targeted therapies may be more beneficial when given prior to ICI in this disease. These approaches will need to be systematically evaluated in preclinical models as well as clinical trials for this disease.

CD8^+^ T cells are traditionally considered as the primary cytotoxic effector in cancer. Multiple studies have demonstrated that antitumor CD4^+^ T cell function is broader than serving as a helper cell to CD8^+^ T cell–mediated killing (52). They were shown to have direct cytotoxic activity upon recognition of peptides presented by MHCII (11, 53) and to cooperate with inflammatory myeloid cells to induce cell death (13). We demonstrate a central role for CD4^+^ T cells in mediating the response to oncogene inhibition in *BRAF^V600E^*-driven ATCs, as evidenced by the enrichment of CD4^+^ T cells in the TME in response to this treatment and the attenuated response in the context of CD4^+^ T cell depletion. The loss of tumoral MHCII presentation in recurrent ATCs refractory to BRAF inhibitors implicates CD4^+^ T cell cytotoxicity as a central mechanism of response to this therapy, which is nominally directed against a cell-autonomous driver of the disease. It also points to a critical role of illegitimate MAPK pathway activation in disabling immune surveillance, a process that may be required for disease pathogenesis.

## Methods

### Sex as a biological variable.

For in vivo orthotopic mouse experiments, we exclusively used female mice to maintain consistency. For GEMMs, both male and female mice were included to ensure findings were broadly representative and to allow assessment of potential sex differences.

### Patient population.

After IRB approval 28 patients with confirmed *BRAF^V600E^* ATC and available paraffin embedded tissue were selected for this study. Chart review was performed to identify the MAPK inhibitor treatment regimen and therapy response at the time of tissue collection (Table 1). Patients were either treated with dabrafenib and trametinib (*n* = 22), vemurafenib (*n* = 1), dabrafenib (*n* = 1), the pan-RAF inhibitor PLX8394 (*n* = 1) or did not receive MAPK inhibitor therapy (*n* = 3). Specimens collected at different times during the treatment were available from 4 patients. Specimens were either collected prior to MAPK inhibitor treatment (MAPKi naive), during the treatment with response (MAPKi PR) or with progression (MAPKi PD).

### Multispectral imaging.

MSI was performed by the Vectra 3.0 Automated Quantitative Pathology Imaging System (Perkin Elmer) on tissue microarrays of 41 PTCs, 72 PDTCs, and 16 ATCs. Five-micron sections of the tissue microarrays were sequentially stained for CD8, CD3, CD163, CD68, and CD15 on a Bond RX autostainer (Leica). The antibodies used are listed in Supplemental Table 3. Slides were dewaxed and antigen retrieval was performed with epitope retrieval solution 1 or 2 (ER1/2, Leico Biosystems) for 20 minutes at 93°C. Following a 30-minute block (Antibody Diluent reagent; Perkin Elmer), tissues were incubated for 30 minutes with the primary antibody, 10 minutes with HRP-conjugated secondary polymer (anti-mouse/anti-rabbit, Perkin Elmer), and 10 minutes with HRP-reactive OPAL fluorescent reagents (Perkin Elmer). Slides were washed between staining steps with Bond Wash (Leica) and stripped between each round (ER1/2). After the final staining, slides were heat-treated (ER1), stained with DAPI (Perkin Elmer), and cover slipped with Prolong Diamond mounting media (Thermo Fisher). Each tissue plug on the microarray was imaged at 20× and analyzed as a single region of interest (ROI). Images were analyzed with inForm software (Perkin Elmer) to unmix fluorochromes, subtract autofluorescence, segment tissue tumor and stromal regions, segment cellular compartments, and phenotype the cells according to morphology and cell marker expression. Cell phenotypes were defined as: CD8^+^ T cell: CD8^+^CD3^+^; “M1-like” macrophage: CD68^+^CD163^–^; “M2-like” macrophage: CD68^+^CD163^+^; and PMN-MDSC: CD15^+^.

### HLA-DR immunohistochemistry.

Slides were incubated with HLA-DR antibody (Supplemental Table 3) at a dilution of 1:500 for 30 minutes. The percentage of tumor cells showing membranous immunopositivity of HLA-DR was recorded.

### ATC mouse models.

We generated a murine ATC model by crossing *Tpo-Cre* (54), *LSL-eYFP* (Jackson Laboratory; stock number 007903), *BRaf-CA* (55) (Jackson Laboratory; stock number: 017837) and *Trp53^fl,fl^* (56) (Jackson Laboratory; stock number: 008462) mice to create quadruple *Tpo-Cre/eYFP/BRaf-CA/Trp53^fl/fl^* transgenics, herein referred as Braf-CA^V600E^/p53 ATC, all alleles were backcrossed into the C57BL/6J background (Jackson Laboratory; stock number: 000664). These multitransgenic mice result in Tpo-Cre driven thyroid-specific expression of BrafV600E, loss of p53 and expression of YFP. Additionally, we generated *Tpo-Cre/eYFP/BRaf-CA* that served as a PTC control (Braf-CA^V600E^ PTC). The *Tpo-Cre/Lsl-rtTA_GFP/tetO-mycBRAFV600E/Trp53^fl/f^* GEMM referred as BRAF/p53 in this work was previously described (26). For the induction of thyroid-specific expression of BRAFV600E mice were fed dox-impregnated chow (2,500 ppm, Envigo). Orthotopic ATCs (Braf/p53) were generated by ultrasound guided injection of 5 μL PBS containing 50,000 TBP3743 cells (30) into the right thyroid lobe of F1 B6129SF1/J mice, purchased from Jackson Laboratory. Briefly, mice were anesthetized by inhalation of ~2% isoflurane with ~2% O_2_ and neck hair was removed using defoliating agent. Orthotopic tumor growth was monitored by weekly ultrasound.

### Mouse imaging studies.

For ultrasound imaging mice were anesthetized by inhalation of 1.5%–2.5% isoflurane with 2% O_2_. An aqueous ultrasonic gel was applied on the denuded neck and thyroid tumors were imaged with the VisualSonics Vevo 770 In Vivo High-Resolution Micro-Imaging System (VisualSonics Inc, Toronto, Ontario, Canada). Using the Vevo 770 scan module, the entire thyroid bed was imaged with captures every 250 microns. Using the instrument’s software, the volume was calculated by manually tracing the margin of the tumor every 250 microns. MRI of thyroid tumors from the *Tpo-Cre/LSL-rtTA_GFP/tetO-mycBRAF*^V600E^ *Trp53^fl/fl^* genetic engineered mice was done as described (26).

### In vivo drug studies.

Treatment with 30 mg/kg dabrafenib (Selleck Chemical) and 3mg/kg trametinib (Selleck Chemical) Monday to Friday via oral gavage was initiated at least 7 days after orthotopic injection of the TBP3743 cell line. For CD4^+^ and CD8^+^ T cell depletion studies, treatment was initiated 1 day prior to tumor implantation with 200 μg of either αCD4 (clone GK1.5, BioXCell) or αCD8 (clone 2.43, BioXCell) and consequently 3×/week for the duration of the study. Tumor growth was assessed by serial ultrasounds.

### Mouse H&E and immunofluorescence.

Mice were euthanized with CO_2_ according with institutional guidelines. Thyroid tumors were harvested and fixed in 4% paraformaldehyde, embedded in paraffin, sectioned, and stained with hematoxylin and eosin (H&E) by the MSK Molecular Cytology Core Facility. Automated multiplex IF was conducted with the Leica Bond BX staining system. Paraffin-embedded tissues were sectioned at 5 μm and baked at 58°C for 1 hour. Slides were loaded in Leica Bond and IF staining was performed as follows: Samples were dewaxed and pretreated with EDTA-based epitope retrieval ER2 solution (Leica, AR9640) for 20 minutes at 100°C. The multiplex antibody staining, and detection were conducted sequentially. The antibodies are listed in Supplemental Table 3. After each round of IF staining, epitope retrieval was performed for denaturation of primary and secondary antibodies before another primary antibody was applied. Finally, slides were washed in PBS and incubated in 5 μg/mL DAPI (Sigma Aldrich) in PBS for 5 minutes, rinsed in PBS, and mounted in Mowiol 4–88 (Calbiochem). Slides were kept at –20°C.

### Tissue processing for flow cytometry.

Mice were euthanized, and thyroid tumors harvested on ice, chopped with razor blades and incubated in 5 mL FACS Buffer (1× HPSS, 5% FBS) with 1.5 mg/mL Collagenase A (Roche) and 0.6 mg/mL bovine DNAse (Sigma Aldrich) at 37°C for 45 minutes mixing at 200 rcf. Cell suspension is then filtered through 70 μM cell strainers (Falcon) and red blood cell (RBC) lysis (10× RBC lysis buffer, Biolegend).

### Flow cytometry.

Single cell suspensions 1 × 10^6^ cell were stained with fixable live and dead stain (Fixable Live and Dead Blue, Thermo Fisher) in PBS, incubated with Fc Block (CD16/CD32 antibody, NovusBio) followed by extracellular antibody cocktail incubation in Brilliant Stain Buffer (BD Biosciences). Cells were then prepared for intracellular stain by incubation with Foxp3 / Transcription Factor Staining Buffer (Tonbo Biosciences) for 1 hour. Afterward cells were washed and incubated with the intracellular antibody cocktail for 15 minutes. Samples were then washed 3 times in FACS Buffer and subjected to analysis at the 5-L Cytek Aurora instrument. Extracellular and intracellular antibodies are listed in Supplemental Table 3. Analysis was performed using FlowJo Version 10.10.7.

### Cell lines.

The TBP3743 cell line was a gift from Sareh Parangi (30). TBP3743 cells were cultured in DME HG 5% fetal bovine serum (FBS, Omega Scientific) and 1% pen/strep/glutamine (PSG, Gemini Bio Products). B92, B16509, B36244, B36934, B34286, and B34838 were generated as previously described (26) and maintained in F12 Coon’s with 5% FBS and 1% PSG.

### Generation of Ciita and H2-ab1 CrisprKO clones.

For each Ciita- and H2-ab1 CRISPR-Cas9 knockout, clones were created using a guide RNAs targeting 2 distinct sites in exon 10 for Ciita and exon 2 for *H2-ab1*. Gene-targeting dual-guide RNA with Cas9 and mCherry coexpression vectors for each respective KO were custom designed and synthesized by VectorBuilder Inc. (Supplemental Table 4). The Ciita and H2-ab1 CRISPR-Cas9 plasmids were transfected in TBP3743 cells with Lipofectamine 3000 (Invitrogen). Thirty-six hours after transfection, cells were FACS sorted based on positive mCherry fluorescence, and single-cell clones were isolated, and the gRNA-targeted region was screened by PCR (Supplemental Table 5) to confirm CRISPR knockouts.

### Trametinib dose-response curves.

Parental, *Ciita^–/–^* or *H2-Ab1^–/–^* TBP3743 cells were plated in Ultra-Low Adherence 96-well plates and cultured with increasing concentrations of trametinib for 5 days in 5% FBS, 1% Penicillin/Streptomycin/Glutamine and 0.5% methylcellulose. Tumor cell spheroids were then incubated with CellTiter-Glo 3D Cell Viability Assay reagents and quantified in a Promega GloMax 96 Microplate Luminometer. Absolute viability values were converted to percentage viability as compared with DMSO-treated controls. IC_50_ curves were generated with GraphPad Prism V10.0 using nonlinear fit of log (inhibitor) vs. response (3 parameters).

### Western blotting.

Cells were lysed in 1 × RIPA buffer (Millipore) supplemented with protease (Roche) and phosphatase inhibitor cocktails I and II (Sigma). Protein concentrations were estimated by BCA kit (Thermo Scientific) on a microplate reader (SpectraMax M5); comparable amounts of proteins were subjected to SDS-PAGE using NuPAGE 4%–12% Bis–Tris gradient gels (Invitrogen) and transferred to nitrocellulose membranes. After overnight application of the primary antibody membranes were incubated with secondary antibodies coupled to horseradish peroxidase (HRP) or IRDye fluorophores for 1 hour at room temperature. ERK and STAT blots were imaged using iBright CL1000 (Thermo Fisher Scientific). For EZH2, H3 and H3K37Me3 imaging was done using the LI-COR Odyssey. Western blot antibodies are listed in Supplemental Table 3.

### Real-time PCR.

One microgram of RNA was subjected to DNase I (Invitrogen) treatment and reverse transcribed using SuperScript III Reverse Transcriptase (Invitrogen) following the manufacturer’s protocol. cDNA was diluted at 1:10 and 2 μL used as a template for RT-PCR reactions performed using the Power SYBR Green PCR Master Mix (Applied Biosystems) on QuantStudio 8 pro (Applied Biosystems). For gene expression quantifications the Ct values of the target genes were normalized to β-actin. The PCR primers are listed in Supplemental Table 5.

### Bulk transcriptomic sequencing.

For sorted tumor cells from the GEMM, 1–2 ng total RNA quantified with RiboGreen with RNA integrity numbers ranging from 7.2 to 10 underwent amplification using the SMART-Seq v4 Ultra Low Input RNA Kit (Clonetech catalog # 63488), with 12 cycles of amplification. Subsequently, 9–10 ng of amplified cDNA was used to prepare libraries with the KAPA Hyper Prep Kit (Kapa Biosystems KK8504) using 8 cycles of PCR. Samples were barcoded and run on a HiSeq 4000 in a PE50 run, using the HiSeq 3000/4000 SBS Kit (Illumina). An average of 53 million paired reads were generated per sample and the percent of mRNA bases per sample ranged from 65% to 78% and ribosomal reads averaged 1%. For the sorted tumor cells from orthotopic ATCs 1 μg of total RNA with DV200 percentages varying from 49%–90% underwent ribosomal depletion and library preparation using the TruSeq Stranded Total RNA LT Kit (Illumina catalog # RS-122-1202) according to instructions provided by the manufacturer with 8 cycles of PCR. Samples were barcoded and run on a NovaSeq 6000 in a PE100 run, using the NovaSeq 6000 S4 Reagent Kit (200 Cycles) (Illumina). On average, 34 million paired reads were generated per sample and 60% of the data mapped to the transcriptome.

### ATAC sequencing.

Profiling of chromatin was performed by ATAC-Seq as described (57). Briefly, ~50,000 cells were washed in cold PBS and lysed. The transposition reaction containing TDE1 Tagment DNA Enzyme (Illumina catalog # 20034198) was incubated at 37°C for 30 minutes. The DNA was cleaned with the MinElute PCR Purification Kit (QIAGEN catalog # 28004) and material amplified for 5 cycles using NEBNext High-Fidelity 2X PCR Master Mix (New England Biolabs catalog # M0541L). After evaluation by real-time PCR, 9–11 additional PCR cycles were done. The final product was cleaned by aMPure XP beads (Beckman Coulter catalog # A63882) at a 1× ratio, and size selection was performed at a 0.5× ratio. Libraries were sequenced on a NovaSeq 6000 in a PE100 run, using the NovaSeq 6000 S4 Reagent Kit (200 Cycles) (Illumina). An average of 36 million paired reads were generated per sample.

### Whole exome capture and sequencing.

After PicoGreen quantification and quality control by Agilent BioAnalyzer, 100 ng of DNA were used to prepare libraries using the KAPA Hyper Prep Kit (Kapa Biosystems KK8504) with 8 cycles of PCR. After sample barcoding, 340–500 ng of library were captured by hybridization using the SinglePlex Mouse Exome (Twist catalog # 102036) according to the manufacturer’s protocol. PCR amplification of the post-capture libraries was carried out for 12 cycles. Samples were run on a NovaSeq 6000 in a PE100 run, using the NovaSeq 6000 S4 Reagent Kit (200 Cycles) (Illumina). Depth of sequencing averaged 78X.

### Bioinformatic analysis for WES.

Illumina (HiSeq) Exome Variant Detection Pipeline: The data processing pipeline for detecting variants in Illumina HiSeq data are as follows. First the FASTQ files are processed to remove any adapter sequences at the end of the reads using cutadapt (v1.6). The files are then mapped using the BWA mapper (bwa mem v0.7.12). After mapping the SAM files are sorted and read group tags are added using the PICARD tools. After sorting in coordinate order, the BAM’s are processed with PICARD MarkDuplicates. The marked BAM files are then processed using the GATK toolkit (v 3.2) according to the best practices for tumor normal pairs. They are first realigned using ABRA (v 0.92) and then the base quality values are recalibrated with the BaseQRecalibrator. Somatic variants are then called in the processed BAMs using muTect (v1.1.7) for SNV and the Haplotype caller from GATK with a custom post-processing script to call somatic indels. The full pipeline is available at https://github.com/soccin/BIC-variants_pipeline and the post-processing code is at https://github.com/soccin/Variant-PostProcess Data available under accession GSE301723.

### RNA-Seq analysis.

Raw sequencing reads were 3’ trimmed for quality <15 and adapters using version 0.4.5 of TrimGalore (https://www.bioinformatics.babraham.ac.uk/projects/trim_galore), and then aligned to mouse assembly mm9 with STAR v2.4 using default parameters. Post-alignment quality and transcript coverage were assessed using the Picard tool CollectRNASeqMetrics (http://broadinstitute.github.io/picard/). Raw read count tables were created using HTSeq v0.9.1. Normalization and expression dynamics were evaluated with DESeq2 using the default parameters including library size factor normalization. Heat maps were created using z-transformed normalized counts and plotted using pheatmap in R. Data available under accession GSE302631.

### Epigenomic analysis.

ATAC sequencing reads were 3’ trimmed and filtered for quality and adapter content using version 0.4.5 of TrimGalore, with a quality setting of 15, and running version 1.15 of cutadapt and version 0.11.5 of FastQC. Reads were aligned to mouse assembly mm9 with version 2.3.4.1 of bowtie2 (http://bowtie-bio.sourceforge.net/bowtie2/index.shtml) and were deduplicated using MarkDuplicates in version 2.16.0 of Picard Tools. To ascertain enriched regions, MACS2 (https://github.com/taoliu/MACS) was used with a *P* value setting of 0.001. A global peak atlas was created by first removing blacklisted regions (http://mitra.stanford.edu/kundaje/akundaje/release/blacklists/mm9-mouse/mm9-blacklist.bed.gz), then defining a peak as ± 250 bp around peak summits, and finally counting reads with version 1.6.1 of featureCounts (http://subread.sourceforge.net). Comparison of intra vs. intergroup clustering in PCA was used to determine normalization strategy, using either the median ratio method of DESeq2 or a sequencing depth-based factor normalized to 10 million uniquely mapped fragments. The BEDTools suite (http://bedtools.readthedocs.io) was used to create normalized read density profiles based on the DESeq2 size factors. Differential enrichment was scored using DESeq2 for all pairwise group contrasts. All differential peaks were then merged for all contrasts in a given dataset, and k-means clustering was performed from k = 4 to the point at which cluster groups became redundant. Peak-gene associations were created using linear genomic distance to TSS. GSEA for epigenomic data was performed with the pre-ranked option and default parameters, where each gene was assigned the single peak with the largest (in magnitude) log_2_ fold change associated with it. Motif signatures were obtained using Homer v4.5 (http://homer.ucsd.edu). Composite and tornado plots were created using deepTools v3.3.0 by running computeMatrix and plotHeatmap on normalized bigwigs with average signal sampled in 25 bp windows and flanking region defined by the surrounding 2 kb. Data available under accession GSE302631.

### Statistics.

Statistical analyses were performed using GraphPad Prism version 10. The choice of statistical tests was based on sample size, data distribution, and experimental design. For comparisons between 2 independent groups, 2-tailed Mann-Whitney *U* tests were applied. This nonparametric test was selected due to small sample sizes and nonnormal data distributions. When multiple such 2-group comparisons were made independently, this was referred to as “multiple Mann-Whitney tests.” For datasets involving 3 or more independent groups, the Kruskal-Wallis test was used. This nonparametric test was chosen because of potential non-Gaussian distributions and unequal variances between groups. Significant Kruskal-Wallis results were followed by appropriate post hoc pairwise comparisons. When assumptions of normality and equal variance were met for comparisons across multiple groups, 2-sided ANOVA with Šidák’s multiple-comparison correction and single pooled variance was performed for family-wise error rate control. Unless stated otherwise, all tests were 2-sided. Data are presented as mean ± SEM. A *P* value less than 0.05 was considered statistically significant.

### Study approval.

All animal studies were conducted in accordance with protocols reviewed and approved by the IACUC of Memorial Sloan Kettering Cancer Center. The studies involving humans were approved by the Memorial Sloan Kettering Cancer Center IRB in New York City using the #12-245 (Genomic Profiling in Cancer Patients) consent form. Written informed consent was received from all study participants.The full data pipeline is available at https://github.com/soccin/BIC-variants_pipeline; commit ID: 88967b8ddc1af03f95f09458d6c2d2073d5bed62 and the post-processing code is at https://github.com/soccin/Variant-PostProcess; commit ID: 8e72acb84500719afde95dcfcfd3b910c59127d2.

### Data availability.

The data necessary to replicate the findings of this study, with the exception of those shown in Figure 3A (OncoPrint based on human biological specimens), are publicly available. Raw and processed data from murine samples have been deposited in the Gene Expression Omnibus (GEO) database: WES data files, GSE301723; ATAC-seq and RNA-Seq data files, GSE302631. Values for all data points are reported in the Supporting Data Values file.

## Author contributions

Conceptualization including ideas and research aims: VT, JAK, and JAF. Experimental design and creation of models: VT, JJ, GPK, JAK, and JAF. Computational analysis: VT, JAK, RPK, and NDS. Data analysis: VT, JDF, BX, and RAG. Data generation: VT, JG, TQ, SYI, JAK, LB, EJS, EDS, and ALH. Manuscript preparation: VT and JAF. Supervision: VT and JAF.

## Supplementary Material

Supplemental data

Unedited blot and gel images

Supplemental table 2

Supporting data values

## Figures and Tables

**Table 1:**
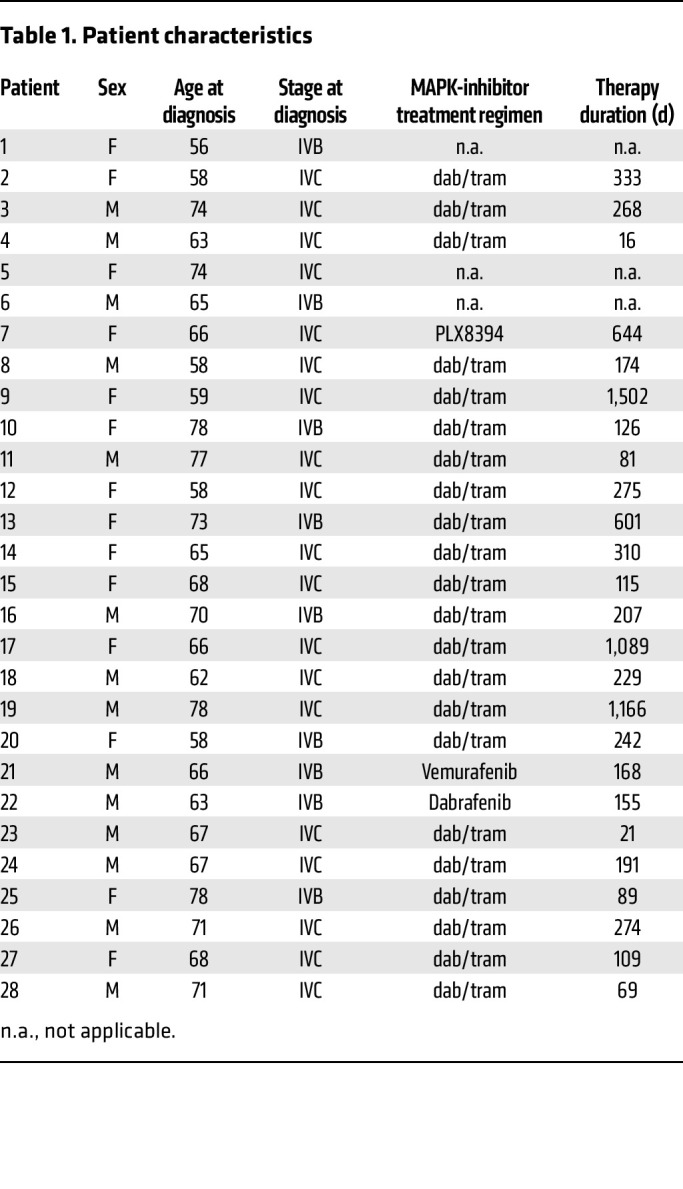
Patient characteristics

## Version 1 08/19/2025

In-Press Preview

## Version 2 10/15/2025

Electronic publication
