# Supplementary material for: Loss of tumor cell MHC class II drives MAPK inhibitor insensitivity of BRAF-mutant anaplastic thyroid cancers
Source: J Clin Invest. 2025 Aug 19;135(20):e191781. doi: 10.1172/JCI191781 (PMC12520682; doi:10.1172/JCI191781)
Supplement: Supplemental data [file jci-135-191781-s314.pdf]

Supplementary Material

Loss of tumor cell MHC Class II drives MAPK-inhibitor insensitivity of BRAF-mutant anaplastic thyroid cancers

Vera Tiedje[1], Jillian Greenberg[1], Tianyue Qin[1], Soo-Yeon Im[1], Gnana P. Krishnamoorthy[1], Laura Boucai[2], Bin Xu[3], Jena D. French[4,5], Eric J Sherman[2], Alan L Ho[2], Elisa de Stanchina[6], Nicholas D. Socci[7], Jian Jin[8], Ronald A. Ghossein[3], Jeffrey A. Knauf[1,9], Richard P Koche[10], James A. Fagin[1,2]

[1] Human Oncology and Pathogenesis Program, Memorial Sloan Kettering Cancer Center, New York, New York, USA.

[2] Department of Medicine, Memorial Sloan Kettering Cancer Center (MSKCC), New York, 10065, USA.

[3] Department of Pathology and Laboratory Medicine, Memorial Sloan Kettering Cancer Center, New York, NY, USA.

[4] Department of Medicine, Division of Endocrinology, Metabolism, and Diabetes, University of Colorado Denver, Aurora, CO, USA. jena.french@CUanschutz.edu.

[5] University of Colorado Cancer Center, University of Colorado Denver, Aurora, CO, USA.

[6] Antitumor Assessment Core Facility, Memorial Sloan Kettering Cancer Center, New York, New York, USA.

[7] Bioinformatics Core, Memorial Sloan Kettering Cancer Center, New York, NY, USA.

[8] Mount Sinai Center for Therapeutics Discovery, Departments of Pharmacological Sciences, Oncological Sciences and Neuroscience, Tisch Cancer Institute, Icahn School of Medicine at Mount Sinai, New York, New York, USA.

[9] Lerner Research Institute, Cleveland Clinic, Cleveland, Ohio, USA.

[10] Center for Epigenetics Research, Memorial Sloan Kettering Cancer Center, New York, New York, USA.

Corresponding Author:
James A. Fagin
Human Oncology and Pathogenesis Program
Memorial Sloan Kettering Cancer Center
1275 York Avenue, New York, NY 10065.
Phone: +1 646-608-2917
E-mail: faginj@mskcc.org

Conflict of interest statement: The authors declare no conflicts of interest with this work.

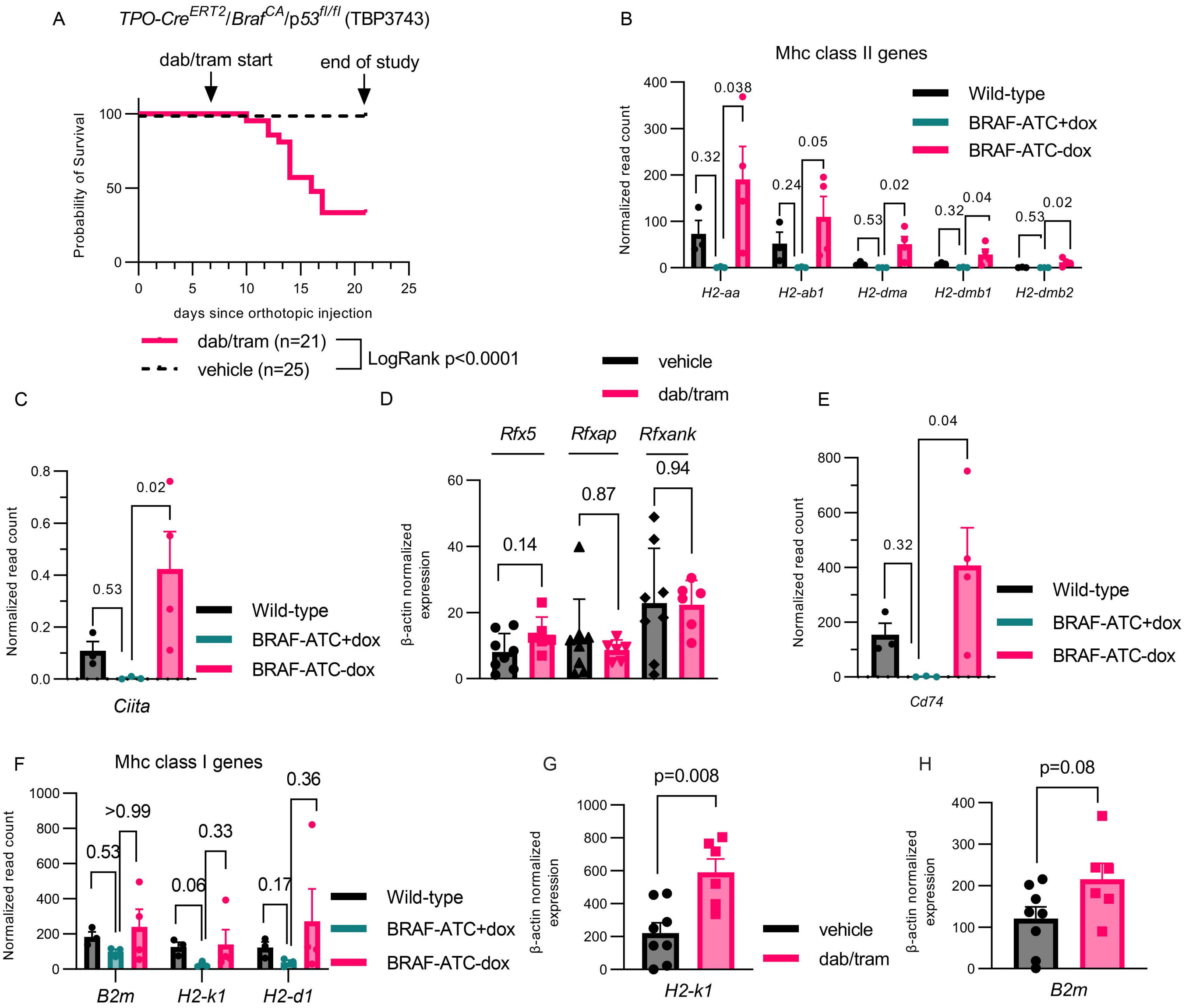

Supplementary Figure 1: A) Kaplan Meier analysis of survival of mice with orthotopic *Braf/p53* ATCs treated with vehicle or dab/tram 1 week after TBP3743 ATC cell injection. B) Expression of MhcII complex genes is low or absent in WT thyrocytes, low in BRAF/p53-ATC cells and induced by dox withdrawal. C) *Ciita* expression in WT thyrocytes and in ATC cells prior to or after dox withdrawal. D) Quantitative RT-PCR of the MhcII-related transcription factors Rfx5, Rfxap and Rfxank is not impacted by dab/tram treatment in the orthotopic Braf/p53 model. E) *Cd74* expression in WT thyrocytes and in ATC cells prior to or after dox withdrawal. F) Expression of MhcI genes in WT thyrocytes or ATC cells prior to or after dox withdrawal. G and H) RT-PCR of H2-k1 and B2m mRNAs in sorted thyrocytes from Braf/p53 orthotopic ATCs treated with vehicle or dab/tram in vivo for 4 days. Log-rank (Mantel-Cox) test (A), Kruskal Wallis Test (B, C, E and F) Multiple Mann-Whitney tests (D, G and H). Bars represent SEM. ATC: Anaplastic thyroid cancer; IFNγ: Interferon γ; GEMM: Genetic engineered mouse model; dab/tram: Dabrafenib and trametinib; Ciita: Class II major histocompatibility complex transactivator; WT: wild type; SEM: Standard error of the mean.

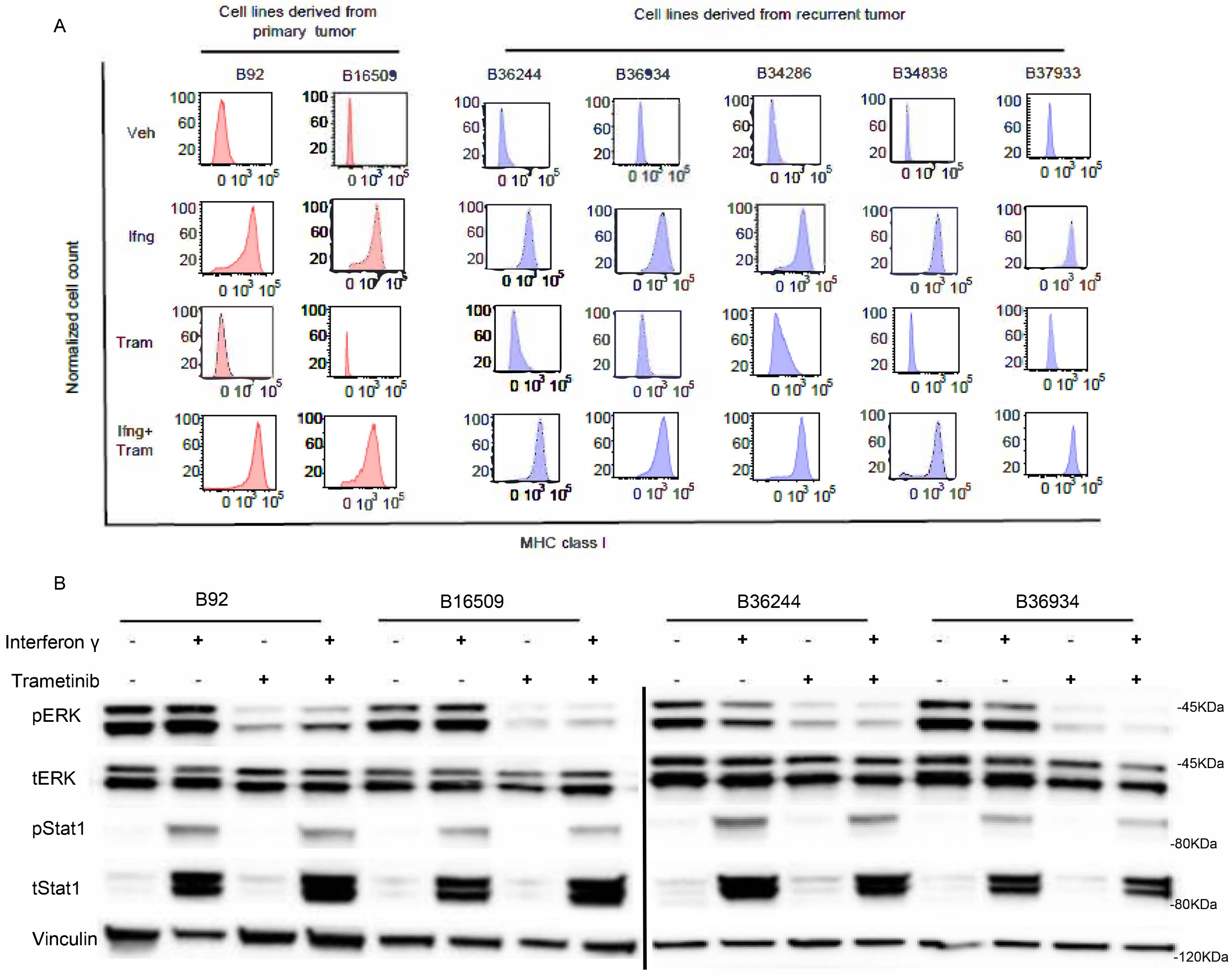

Supplementary Figure 2: A) Induction of MhcI by IFNγ (20ng/ml) as determined by FACS in two primary cell lines derived from primary ATCs on dox diet (B92 and B16509) and five lines derived from recurrent tumors developing after dox withdrawal (B36244, B36934, B34286, B34838 and B37933). Addition of trametinib (10nM) does not significantly augment the IFNγ (20ng/ml) effect. B) Western blot of B92, B16509, B36244, B36934 cell lysates treated with trametinib alone or in combination with IFNγ for 96 h for the indicated proteins. IFNγ: Interferon γ; dox: doxycycline.

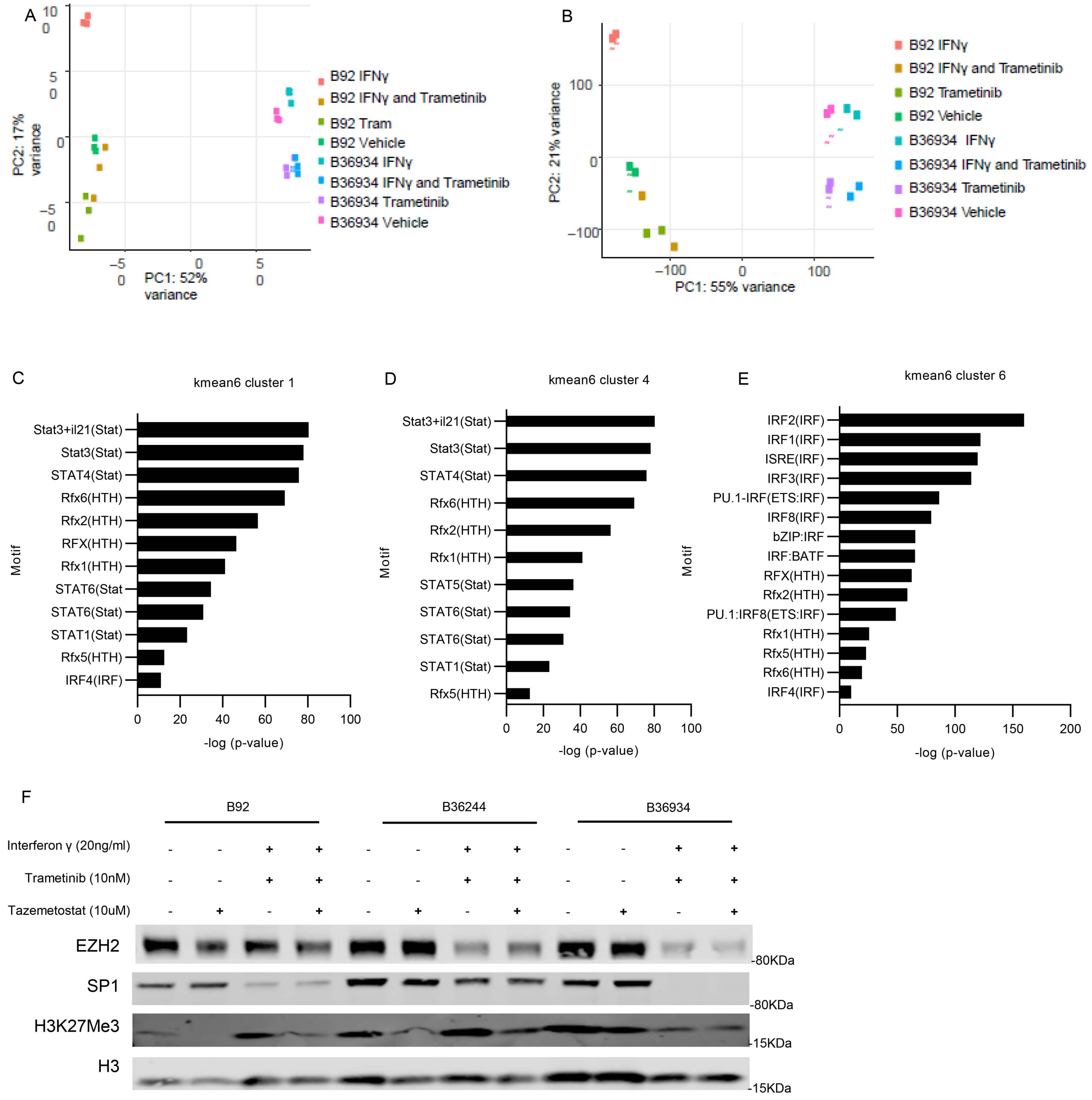

Supplementary Figure 3: A,B) Principal component analyses of RNA Seq and ATAC-Seq for the B92 primary and the B36934 recurrent ATC lines displaying triplicates and duplicates, respectively, for each treatment condition (DMSO, IFNγ (20ng/ml), trametinib (10nM) and the combination of IFNγ + trametinib (10nM) for 96 h. C–E) TF motifs of members of the Rfx, Stat and Irf families enriched in kmeans clusters 1, 4 and 6 identified using HOMER *de novo* motif discovery. F) Western blot for EZH2, Sp1, H3K27Me3 and H3 for the indicated cell lines and treatment conditions. IFNγ: Interferon γ; tram: trametinib; TF: transcription factor.

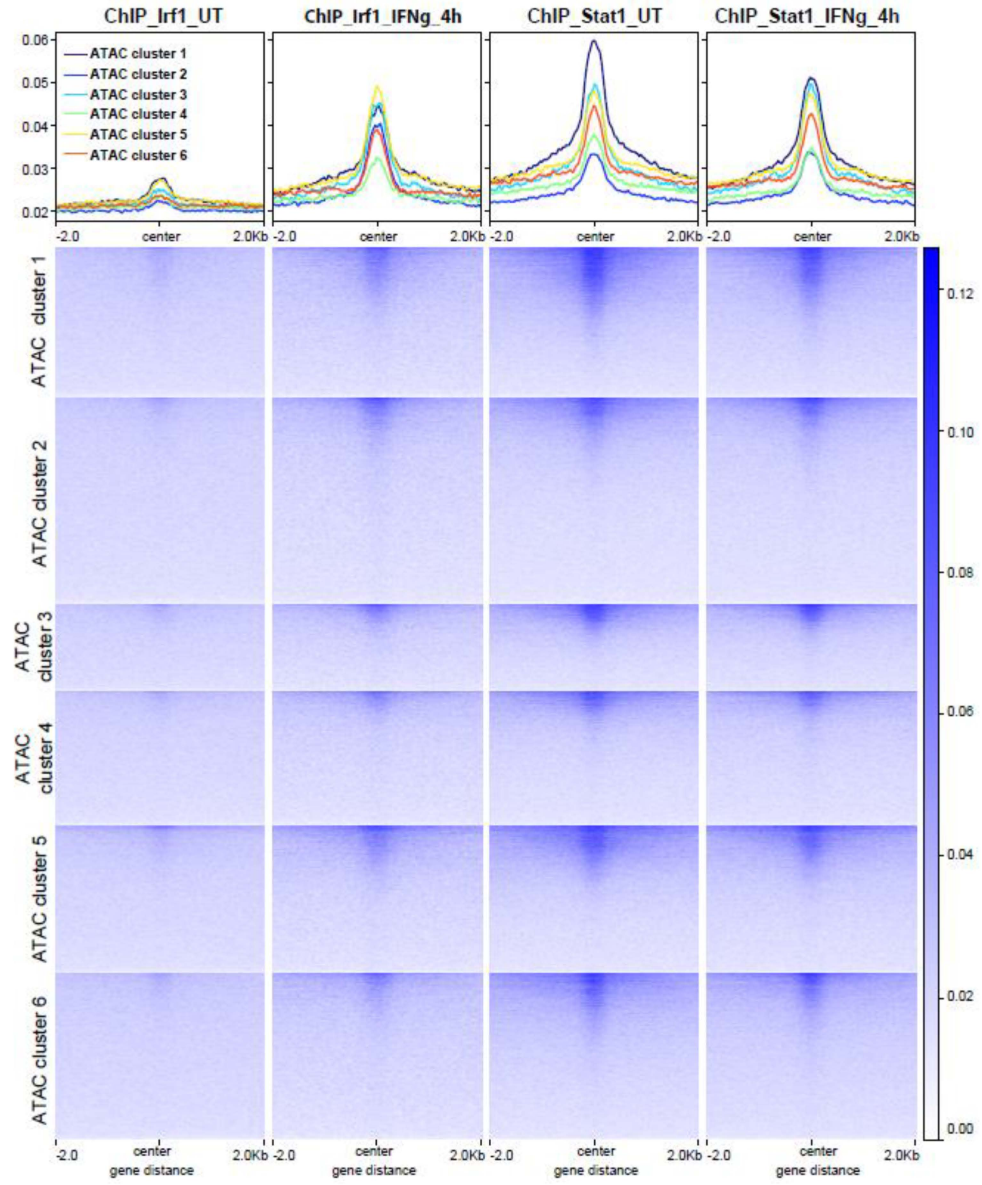

Supplementary Figure 4: Tornado plots of averaged Stat1 and Irf1 transcription factor binding sites in the respective k-means clusters.

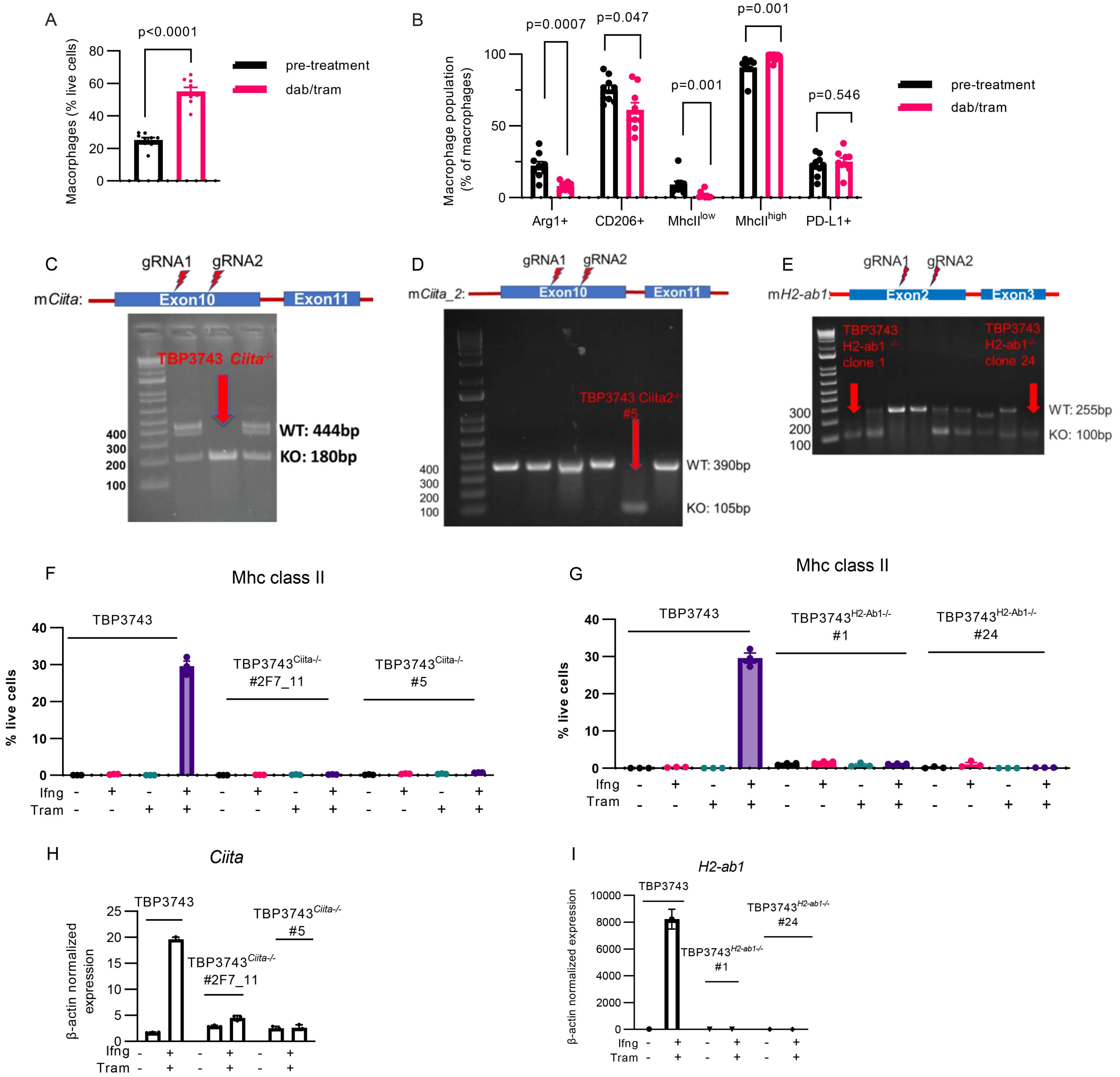

Supplementary Figure 5: A, B) Analysis of infiltrating macrophages by flow cytometry in orthotopic Braf/p53 ATC at baseline and 5 days after treatment with dab/tram. C, D) Top: Schema of two different gRNA pairs targeting Exon 10 for CRISPR KO of Ciita. Bottom: Image of gel electrophoresis of PCR products from the indicated CRISPR KO clones. E) Top: Schema for gRNA pair targeting exon2 of H2-ab1 for generation of CRISPR KO clones. Bottom: Image of gel electrophoresis of PCR products of two homozygous H2-ab1 KO clones. F, G) Loss of IFNγ and trametinib-induced MhcII in the Ciita CRISPR KO clones 2F7_11 and 5 and H2-ab1 CRISPR KO clones 2 and 24, all derived from TBP3743 cells. MhcII was measured by FACS 96h after treatment with the indicated conditions. H, I) Quantitative RT-PCR of Ciita mRNA in parental and Ciita-/- clones and H2-ab1 mRNA in parental and H2-ab1-/- clones. gRNA: guide RNA; IFNγ: Interferon γ; Ciita: Class II major histocompatibility complex transactivator.

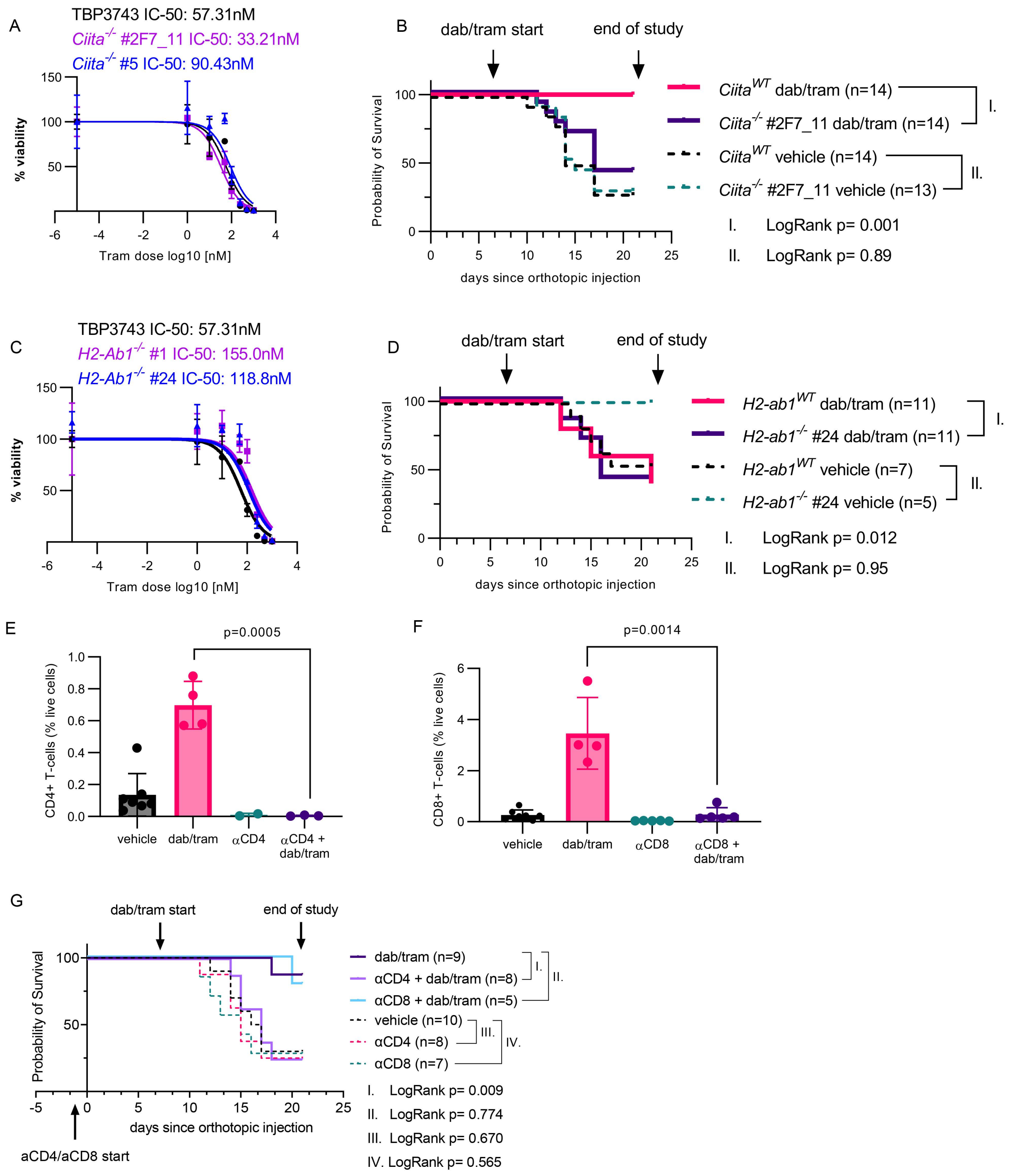

Supplementary Figure 6: A, C) IC50 for trametinib in parental, Ciita-/- clones 2F7_11 and 5 and H2-ab1-/- clones 1 and 24. B, D) Kaplan Meier analysis of survival of mice with orthotopic Braf/p53 ATCs, treated with vehicle or dab/tram for 1 week after orthotopic injection of TBP3743 parental or Ciita-/- or H2-ab1-/- ATC cells. E) and F) CD4+ (E) and CD8+ T-cells (F) percentage of live cells in thyroids harvested from mice treated with in the indication conditions. G) Kaplan Meier analysis of survival of mice with orthotopic Braf/p53 ATCs in the indicated treatment conditions. Tram: trametinib. dab/tram: dabrafenib and trametinib. Ciita: Class II major histocompatibility complex transactivator.

Supplementary Tables

Table 1	Tumor mutational burden in human and murine papillary and anaplastic thyroid cancer
Table 2	Whole exome sequencing results: High confidence Maf
Table 3	Antibodies for IHC, IF, FACS and WB
Table 4	CrisprKO gRNA Seqences
Table 5	Primers

Supplementary Table 1

	Human ATC	Murine ATC
Mutation per tumor	23.7 [1]	22 [3]
	24 [2]	

Reference

[1]Kunstman et al 2015 (28)

[2]Ravi et al 2019 (29)

[3]Knauf et al 2018 (26)

Supplementary Table 3

Antibody	Clone	Source	Target Species
HLA-DR	TAL 1B5	Santa Cruz	human
Pan Cytokeratin	na	Thermo Fisher	human
CD8	4B11	Thermo Fisher	human
CD3	SP7	Abcam	human
CD163	10D6	Abcam	human
CD68	KP1	Abcam	human
CD15	MMA	Abcam	human
BUV395 Rat Anti-Mouse CD45	30-F11	BD Biosciences	mouse
BV510 Rat Anti-CD11b	M1/70	BD Biosciences	mouse
BV750 Rat Anti-Mouse Siglec-F	E50-2440	BD Biosciences	mouse
FITC Rat Anti-Mouse CD3	17A2	BD Biosciences	mouse
BUV805 Rat Anti-Mouse CD8a	53-6.7	BD Biosciences	mouse
BUV661 Mouse Anti-Mouse NK-1.1	PK136	BD Biosciences	mouse
PerCP anti-mouse Ly-6G Antibody	1A8	Biolegend	mouse
Brilliant Violet 711™ anti-mouse CD11c Antibody	N418	Biolegend	mouse
Spark Blue™ 550 anti-mouse I-A/I-E Antibody	M5/114.15.2	Biolegend	mouse
Brilliant Violet 650™ anti-mouse F4/80 Antibody	BM8	Biolegend	mouse
Brilliant Violet 570™ anti-mouse CD4 Antibody	RM4-5	Biolegend	mouse
Spark NIR™ 685 anti-mouse/human CD45R/B220 Antibody	RA3-6B2	Biolegend	mouse
APC-Cy™7 Rat Anti-Mouse Ly-6C	AL-21	BD Biosciences	mouse
Arginase 1 Monoclonal Antibody PerCP-eFluor™ 710[1]	A1exF5	Thermo Fisher	mouse
FOXP3 Monoclonal Antibody eFluor™ 450[1]	FJK-16s	Thermo Fisher	mouse
CD274 (PD-L1, B7-H1) Super Bright™ 436	MIH5	Thermo Fisher	mouse
BV605 Rat Anti-Mouse I-A/I-E	M5/114.15.2	BD Biosciences	mouse
LIVE/DEAD™ Fixable Blue Dead Cell Stain Kit, for UV excitation	na	Thermo Fisher	mouse
Alexa Fluor® 647 anti-mouse CD206[1]	MMR	Biolegend	mouse
PE Mouse Anti-Mouse H-2Kb	AF6-88.5	BD Biosciences	mouse
Phospho-p44/42 MAPK (Erk1/2) (Thr202/Tyr204) (D13.14.4E) XP® Rabbit mAb		Cell Signaling	
p44/42 MAPK (Erk1/2) (L34F12) Mouse mAb		Cell Signaling	
Phospho-Stat1 (Ser727) (D3B7) Rabbit mAb		Cell Signaling	
Stat1 (D1K9Y) Rabbit mAb		Cell Signaling	
Vinculin (E1E9V) XP® Rabbit mAb		Cell Signaling	
SP1 (D4C3) Rabbit mAb		Cell Signaling	
Ezh2 (D2C9) XP® Rabbit mAb		Cell Signaling	
Histone H3 (D1H2) XP® Rabbit mAb		Cell Signaling	
Tri-Methyl-Histone H3 (Lys27) (C36B11) Rabbit mAb		Cell Signaling	

[1] Intracellular antibodies

Supplementary Table 4

Vector name	gRNA Sequence	Source	Identifier
mCiita_1	AGCAGGCCAAGACTTACATG, TAGTCGAGCTGGCCAAGCTG	Vectorbuilder	VB210324-1175xee
mCiita_2	CCCGGAGCCTTAGTCGAGCT, GAGACCCTATGACAACTGG	Vectorbuilder	VB230124-1305eyk
mH2-ab1	ACGGGACGCAGCGCATACGA, GGAGATCCTGGAGCGAACGC	Vectorbuilder	VB230124-1307xfy

Supplementary Table 5

Primer	Sequence
mCiita_Ex3F	ATCTTCCAGCGGAAGCTACTGC
mCiita_Ex3R	CCGGGTTTCTTGCAAGGTGC
mCiita_Ex10F	GGACTCTATGTCAGCCTGCTAGG
mCiita_Ex10R	TGGGCTCGAGGCTGGAAAAC
mH2-ab1_Ex2F	CCGCAGGGCATTTCGTGTAC
mH2-ab1_Ex2R	TCTCCGGCCCCTCGTAGTTGT
mCD74_FW	AAGCAGTGGCTCTTGTTTGAG
mCD74_RV	CTTCCATGTCCAGTGGCTCT
mCiita_FW	AATCTACCACGGTGAGATGCCC
mCiita_RV	TCGGGGAGACTGGGGATACTGA
mH2-aa_FW	GAGCAGCTTCAGAGACCTCC
mH2-aa_RV	CTACGTGGTCGGCCTCAAT
mH2-ab1_FW	CACAGGAGTCAGAAAGGACCTC
mH2-ab1_RV	TGGCAGTCAGGAATTCGGAG
mH2-dma_FW	GAGATTGACCGCTACACGGCAA
mH2-dma_RV	GAAGACAATGCCCATGATGGTGC
mH2-dmb1_FW	AGAGCCTTCTCCAGCGTTTGCA
mH2-dmb1_RV	TGTGGTTTGGGCTACTCGGACA
mH2-dmb2_FW	ACCTTTCTGGGATGTGCTGACC
mH2-dmb2_RV	GTGATGGTCACATCCGCTGGAT
mRfxap_FW	ACGTCAAACTGGAGGAAAGCAC
mRfxap_RV	GAGTAGGTCTTGCAGGGCG
mRfx5_FW	GGAAGACCTTGGTATCCATGCC
mRfx5_RV	GGCTGCTTCTACCAGTTCATCC
Rfxank_FW	GCACATGCCTGTCTGGAAAC
Rfxank_RV	AGCAGGAAGCGAACTGTCTC
Irf1_FW	CAAAGCCACCATGCCAATCACTCG
Irf1_RV	GGCCCAGCTCCGGAACAGACAG
mH2-k1_FW	GGAGCAGGAGGGGCCCGAGTATTG
mH2-k1_RV	CGCCGTCCACGTTTTCAGGTCTTC
mB2m_FW	TCACTGACCGGCCTGTATGCTATC
mB2m_RV	AATGTGAGGCGGGTGGAACTGT
