# Supplementary material for: Loss of tumor cell MHC class II drives MAPK inhibitor insensitivity of BRAF-mutant anaplastic thyroid cancers
Source: J Clin Invest. 2025 Aug 19;135(20):e191781. doi: 10.1172/JCI191781 (PMC12520682; doi:10.1172/JCI191781)

Uncropped/unedited images for Supplementary Figures 2B, 3F, 5C, D and E

Loss of tumor cell MHC Class II drives MAPK-inhibitor insensitivity of BRAF-mutant anaplastic thyroid cancers

Vera Tiedje1, Jillian Greenberg1, Tianyue Qin1, Soo-Yeon Im1, Gnana P. Krishnamoorthy1, Laura Boucai2, Bin Xu3, Jena D. French4,5, Eric J Sherman2, Alan L Ho2, Elisa de Stanchina6, Nicholas D. Socci7, Jian Jin8, Ronald A. Ghossein3, Jeffrey A. Knauf1,9, Richard P Koche10, James A. Fagin1,2

1 Human Oncology and Pathogenesis Program, Memorial Sloan Kettering Cancer Center, New York, NY, USA.
2 Department of Medicine, Memorial Sloan Kettering Cancer Center, New York, NY, USA.
3 Department of Pathology and Laboratory Medicine, Memorial Sloan Kettering Cancer Center, New York, NY, USA.
4 Department of Medicine, Division of Endocrinology, Metabolism, and Diabetes, University of Colorado Denver, Aurora, CO, USA.
5 University of Colorado Cancer Center, University of Colorado Denver, Aurora, CO, USA.
6 Antitumor Assessment Core Facility, Memorial Sloan Kettering Cancer Center, New York, NY, USA.
7 Bioinformatics Core, Memorial Sloan Kettering Cancer Center, New York, NY, USA.
8 Mount Sinai Center for Therapeutics Discovery, Departments of Pharmacological Sciences, Oncological Sciences and Neuroscience, Tisch Cancer Institute, Icahn School of Medicine at Mount Sinai, New York, NY, USA.
9 Lerner Research Institute, Cleveland Clinic, Cleveland, OH, USA.
10 Center for Epigenetics Research, Memorial Sloan Kettering Cancer Center, New York, NY, USA.

Supplementary Figure 2B

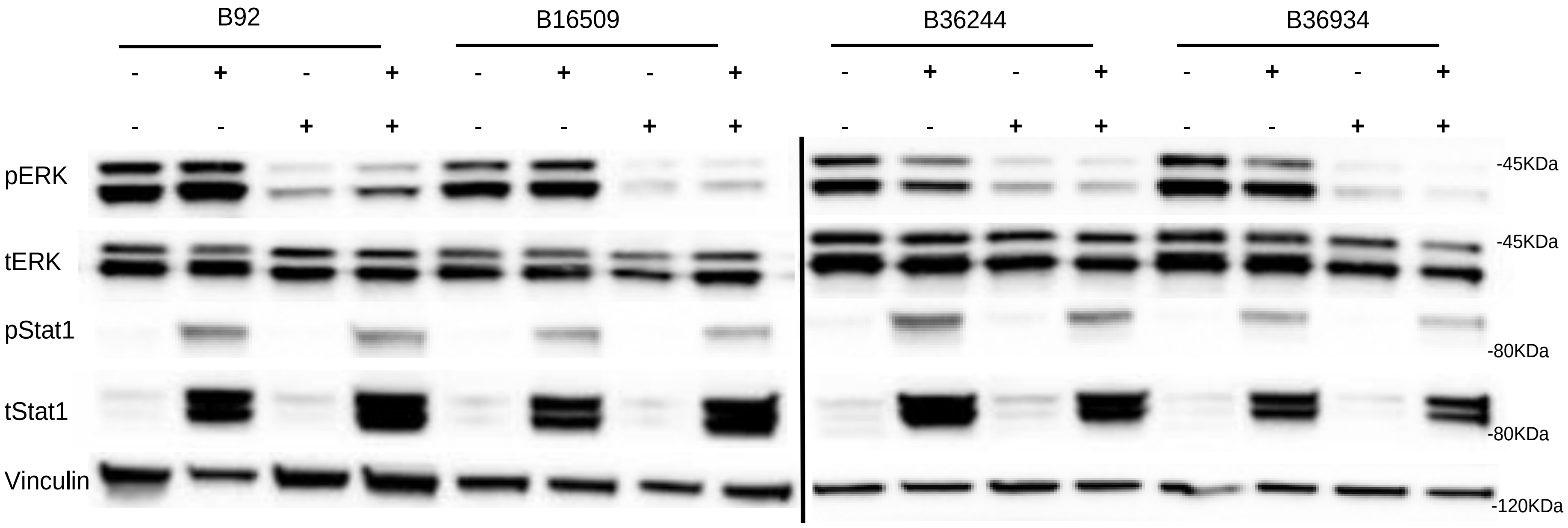

Full unedited gel for Supplementary Figure 2B

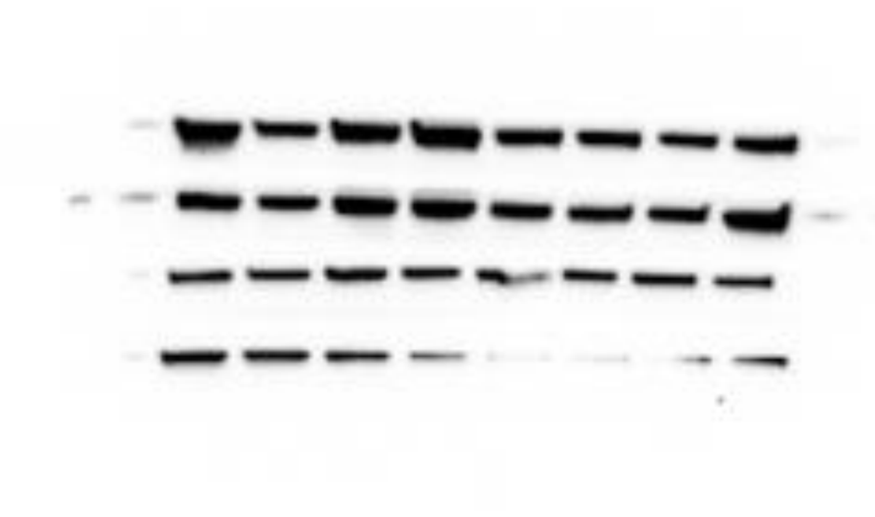

Vinculin B92 and B16509

Vinculin B92 and B16509

Vinculin B36934 and B36244

Vinculin B36934 and B36244

Vinculin (E1E9V) XP® Rabbit mAb

Full unedited gel for Supplementary Figure 2B

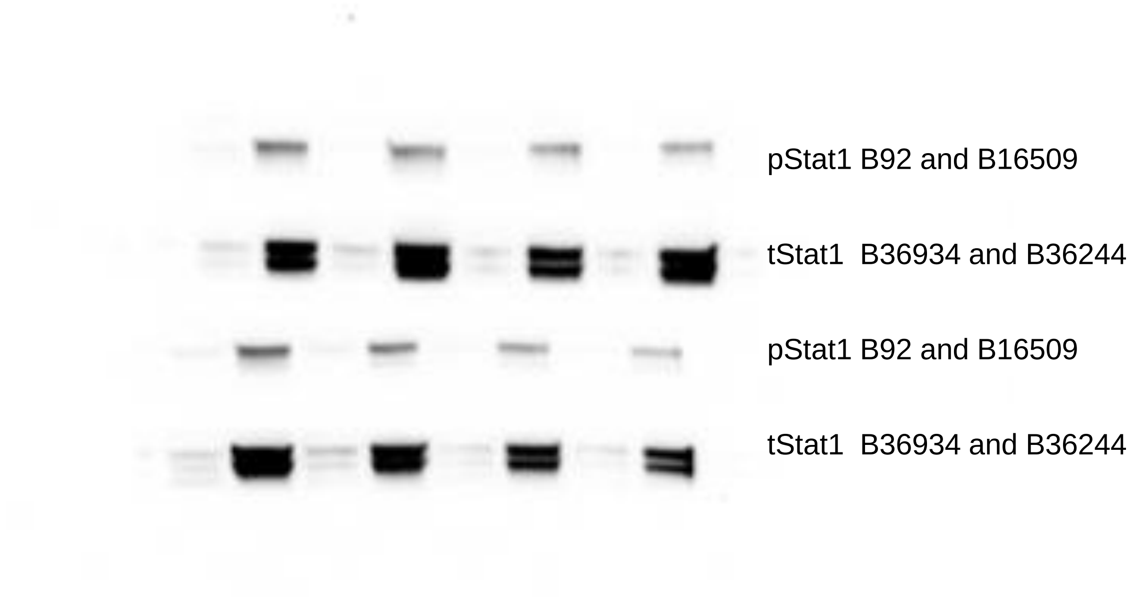

Phospho-Stat1 (Ser727) (D3B7) Rabbit mAb
Stat1 (D1K9Y) Rabbit mAb

Full unedited gel for Supplementary Figure 2B

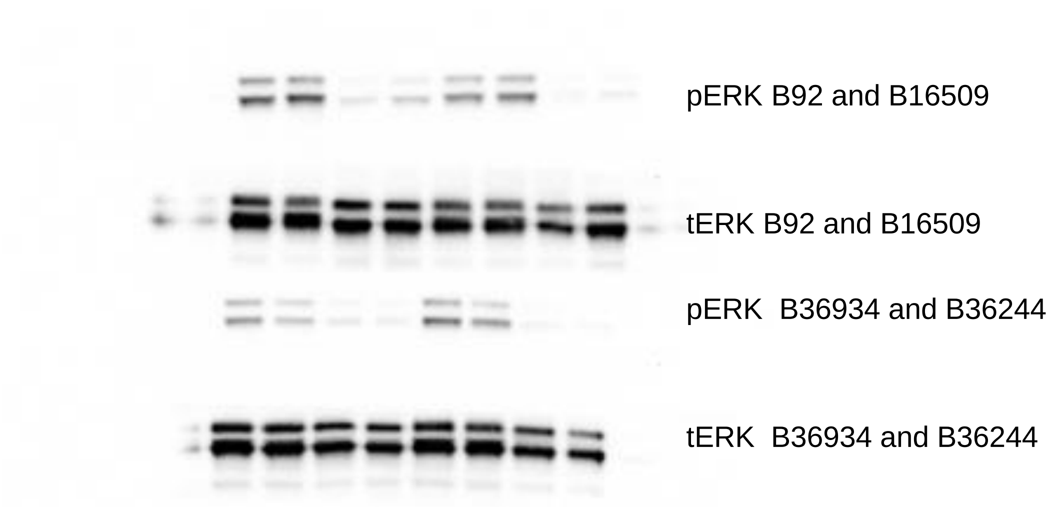

Phospho-p44/42 MAPK (Erk1/2) (Thr202/Tyr204) (D13.14.4E) XP® Rabbit mAb
p44/42 MAPK (Erk1/2) (L34F12) Mouse mAb

Full unedited gel for Supplementary Figure 2B

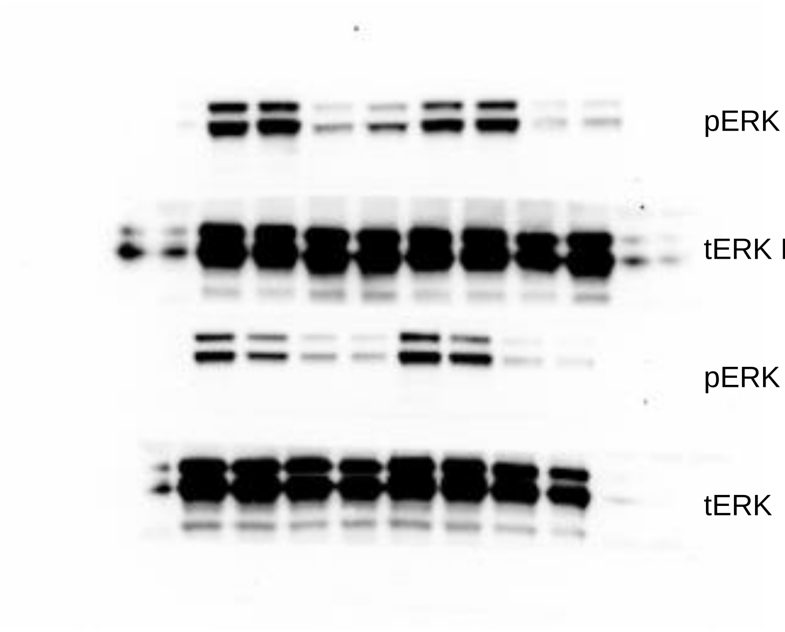

pERK B92 and B16509

tERK B92 and B16509

pERK B36934 and B36244

tERK B36934 and B36244

Phospho-p44/42 MAPK (Erk1/2) (Thr202/Tyr204) (D13.14.4E) XP® Rabbit mAb
p44/42 MAPK (Erk1/2) (L34F12) Mouse mAb

Supplementary Figure 3F

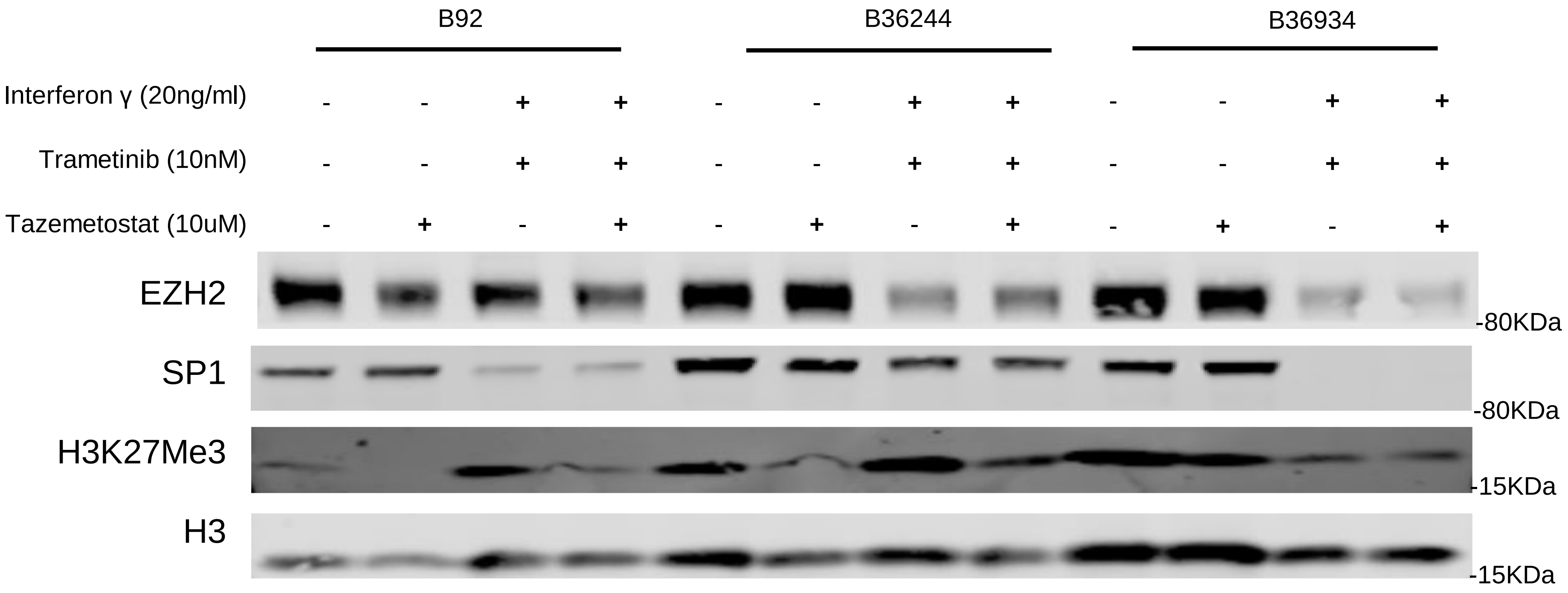

Full unedited gel for Supplementary Figure 3F

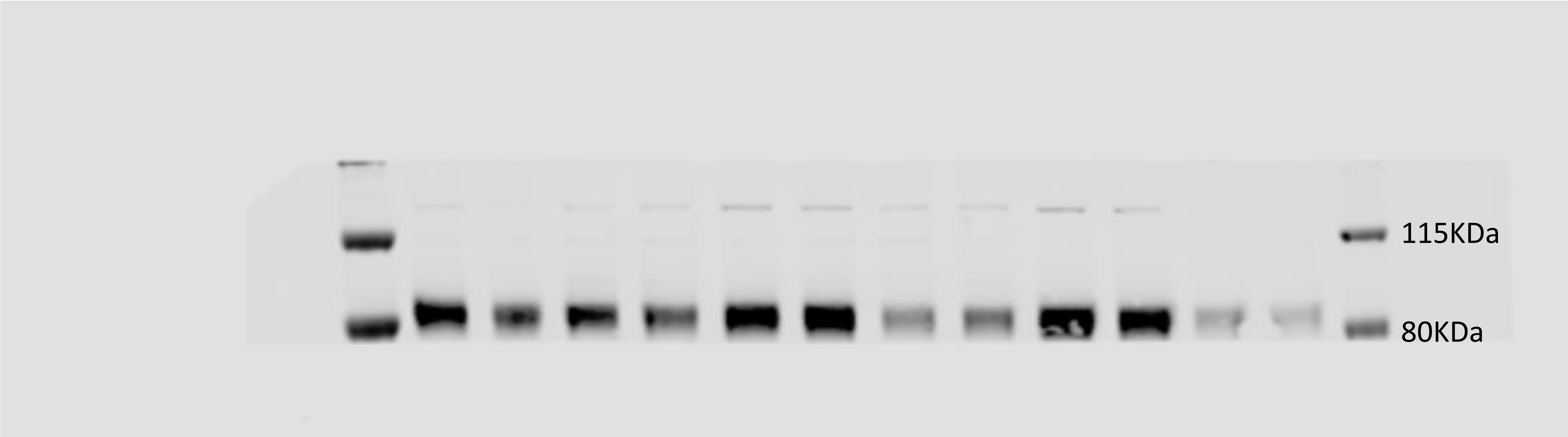

Ezh2 (D2C9) XP® Rabbit mAb

Image is rotated from imagine platform, cut corner represents top left side of membrane

Full unedited gel for Supplementary Figure 3F

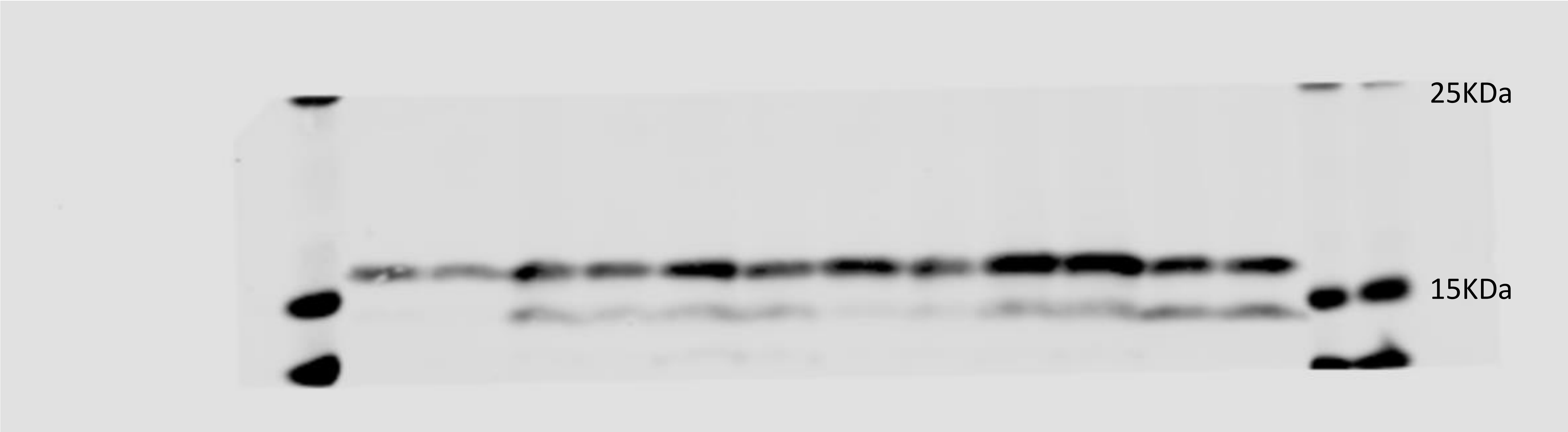

Histone H3 (D1H2) XP® Rabbit mAb

Image is rotated from imagine platform, cut corner represents top left side of membrane.

Full unedited gel for Supplementary Figure 3F

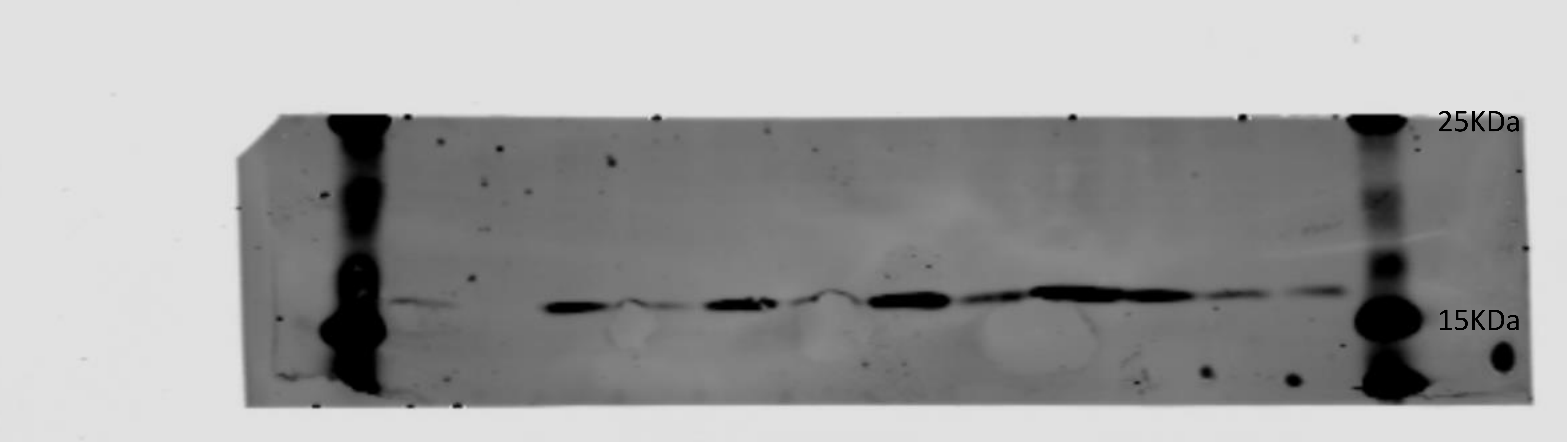

Tri-Methyl-Histone H3 (Lys27) (C36B11) Rabbit mAb

Image is rotated from imagine platform, cut corner represents top left side of membrane.

Full unedited gel for Supplementary Figure 3F

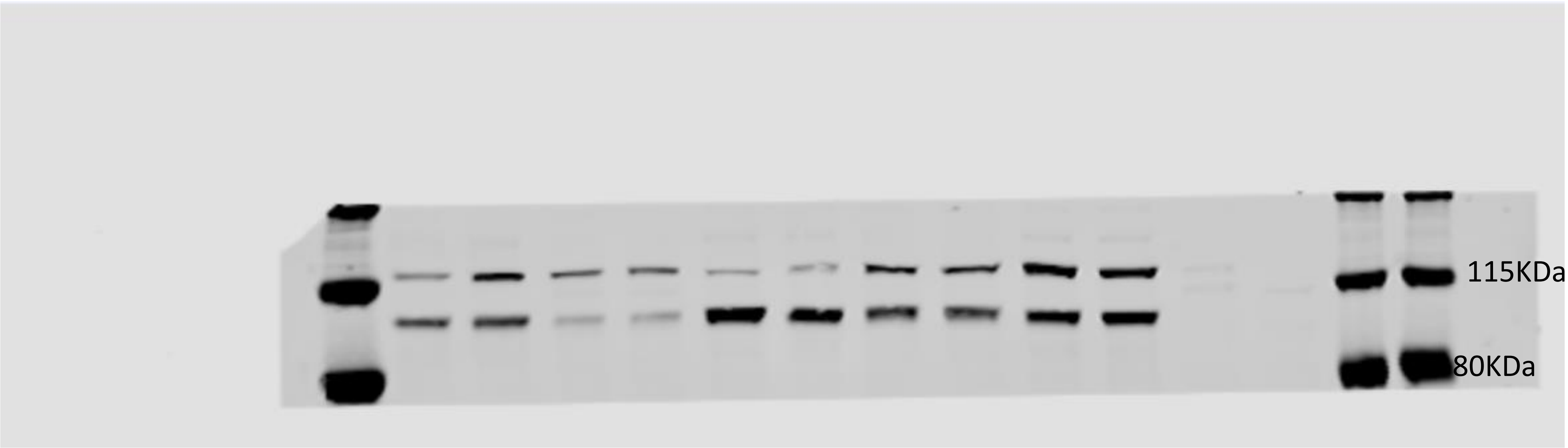

SP1 (D4C3) Rabbit mAb

Image is rotated from imagine platform, cut corner represents top left side of membrane.

Supplementary Figure 5C

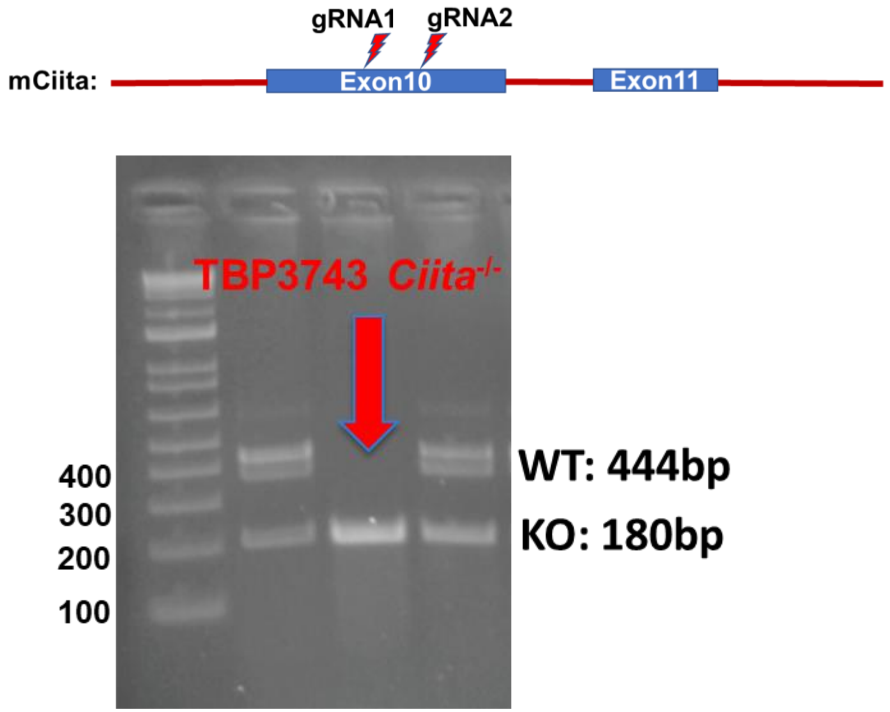

Full unedited picture for Supplementary Figure 5C

TBP3743 *Ciita*$^{-/-}$ #2F7_11

WT:444bp

KO:180bp

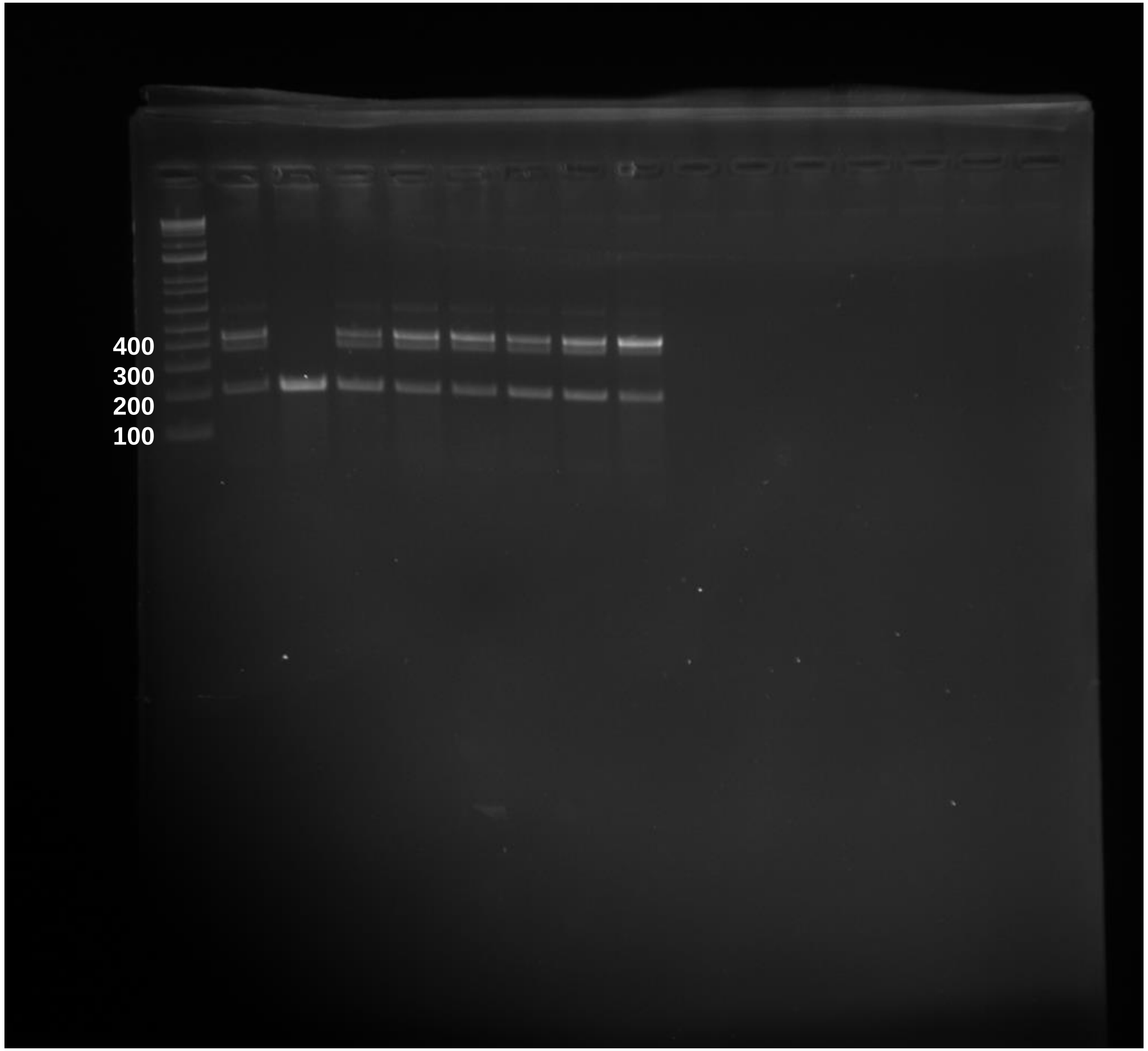

Supplementary Figure 5D

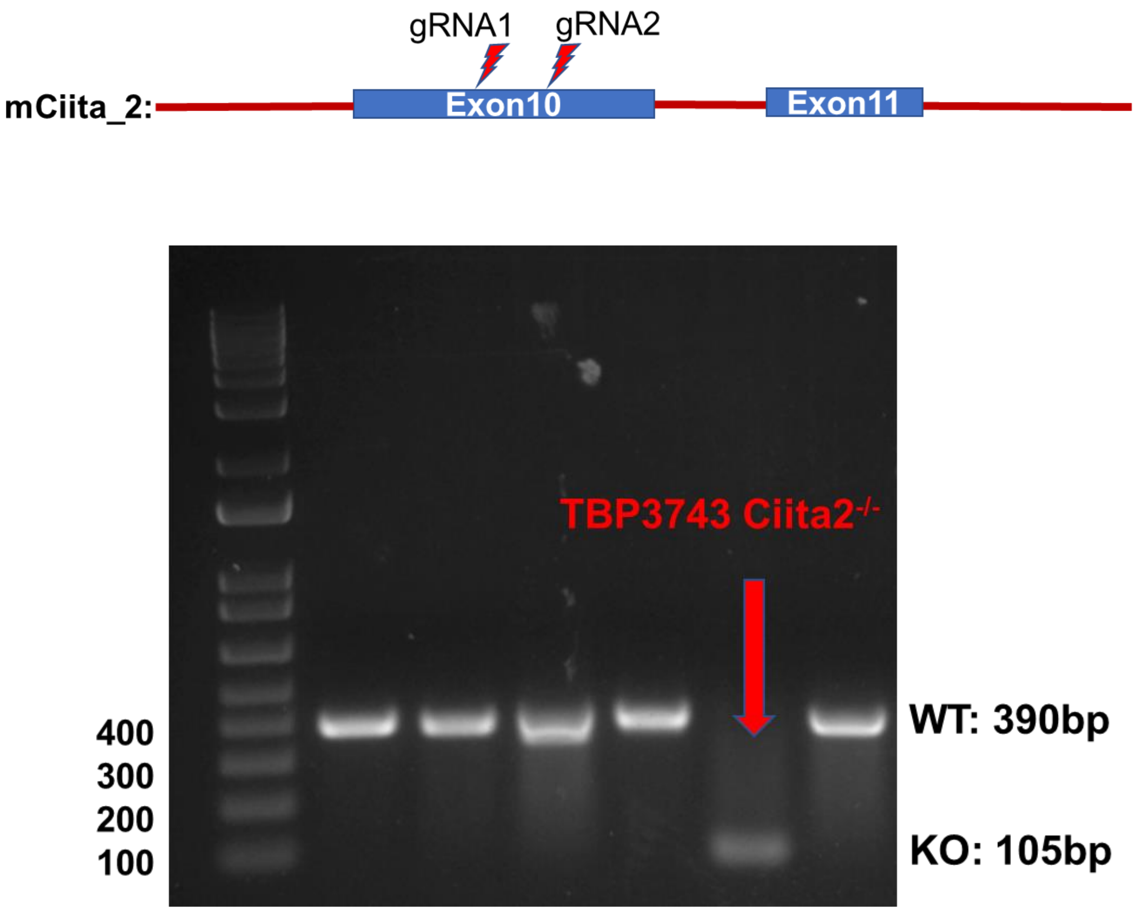

Full unedited picture for Supplementary Figure 5D

TBP3743 *Ciita*$^{-/-}$ _2 #5

WT:390bp

KO:105bp

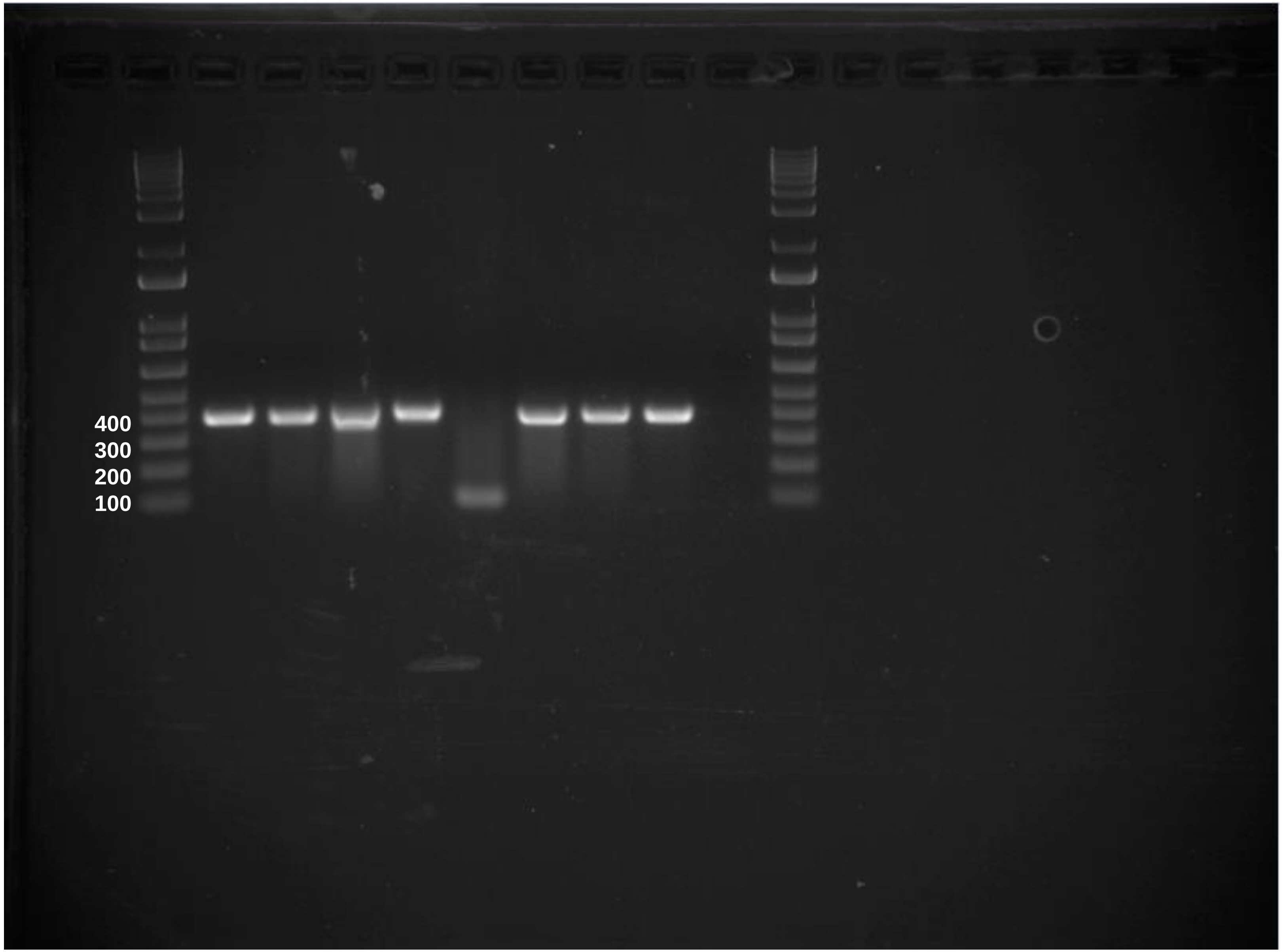

Supplementary Figure 5E

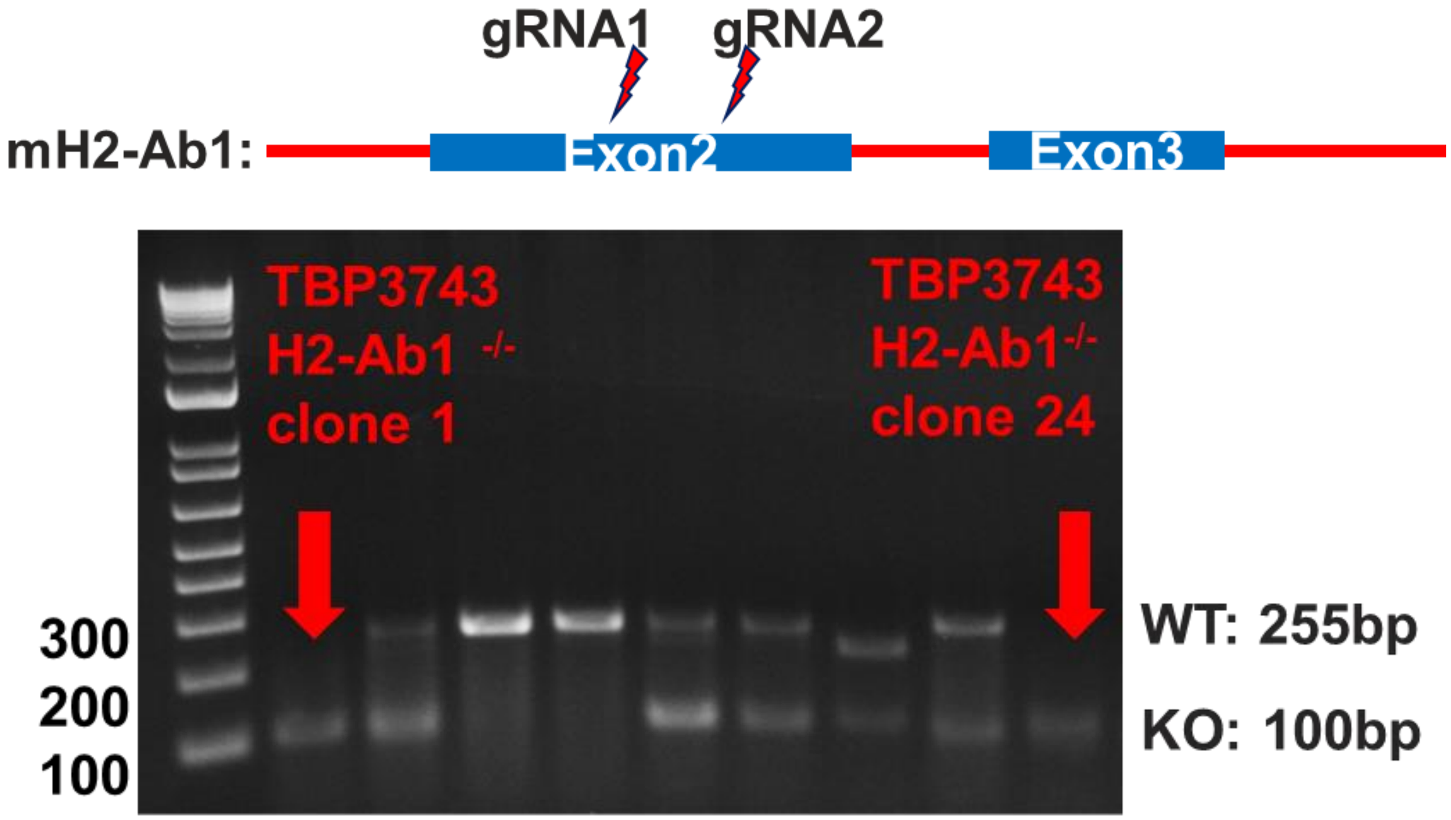

Full unedited picture for Supplementary Figure 5E

TBP3743 *H2-ab1*$^{-/-}$

#1 and #24

WT:255bp

KO:100bp

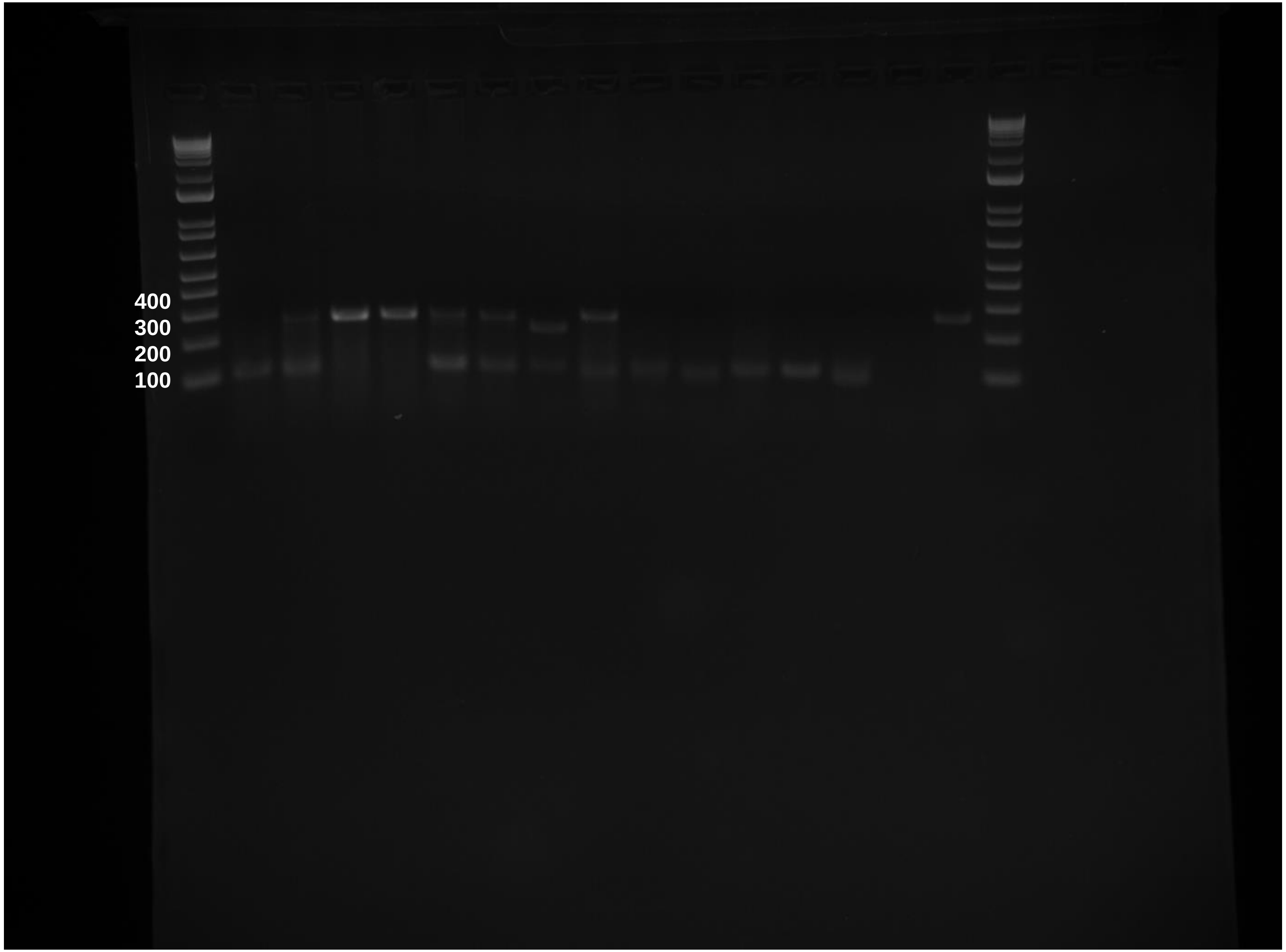

Supplement: Unedited blot and gel images [file jci-135-191781-s315.pdf]
